# A Review on Recent Trends in Bacteriophages for Post-Harvest Food Decontamination

**DOI:** 10.3390/microorganisms13030515

**Published:** 2025-02-27

**Authors:** Márcia Braz, Carla Pereira, Carmen S. R. Freire, Adelaide Almeida

**Affiliations:** 1 CESAM—Centre for Environmental and Marine Studies, Department of Biology, University of Aveiro, Campus Universitário de Santiago, 3810-193 Aveiro, Portugal; marciabraz96@ua.pt (M.B.); csgp@ua.pt (C.P.); 2 CICECO—Aveiro Institute of Materials, Department of Chemistry, University of Aveiro, Campus Universitário de Santiago, 3810-193 Aveiro, Portugal

**Keywords:** foodborne pathogens, post-harvest stage, phage biocontrol, active food packaging, food safety

## Abstract

Infectious diseases resulting from unsafe food consumption are a global concern. Despite recent advances and control measures in the food industry aimed at fulfilling the growing consumer demand for high-quality and safe food products, infection outbreaks continue to occur. This review stands out by providing an overview of post-harvest food decontamination methods against some of the most important bacterial foodborne pathogens, with particular focus on the advantages and challenges of using phages, including their most recent post-harvest applications directly to food and integration into active food packaging systems, highlighting their potential in providing safer and healthier food products. The already approved commercial phage products and the numerous available studies demonstrate their antibacterial efficacy against some of the most problematic foodborne pathogens in different food products, reinforcing their possible use in the future as a current practice in the food industry for food decontamination. Moreover, the incorporation of phages into packaging materials holds particular promise, providing protection against harsh conditions and enabling their controlled and continuous release into the food matrix. The effectiveness of phage-added packaging materials in reducing the growth of pathogens in food systems has been well-demonstrated. However, there are still some challenges associated with the development of phage-based packaging systems that need to be addressed with future research.

## 1. Introduction

Infectious diseases caused by the consumption of contaminated food pose a significant public health threat worldwide. The recurrent use of antibiotics has contributed to the alarming rise in multidrug-resistant bacterial strains and resistance genes, exacerbating this problem. Despite several efforts of the food industry to reduce foodborne pathogens in their products, foodborne infections remain a leading cause of hospitalizations and fatalities on a global scale. According to the World Health Organization (WHO), unsafe food consumption leads to 600 million foodborne illnesses and 420,000 deaths annually, imposing an economic burden exceeding USD 110 billion [1].

*Campylobacter* spp., *Salmonella* spp., Shiga toxin-producing *Escherichia coli* (STEC), and *Listeria monocytogenes* are among the most reported foodborne pathogens in food outbreaks [2]. Other bacteria, such as *Vibrio* spp., *Cronobacter sakazakii*, and *Shigella* spp. have also been reported in a considerable number of cases. *Bacillus cereus*, *Clostridium botulinum*, *Clostridium perfringens*, and *Staphylococcus aureus* are particularly important due to the production of bacterial toxins. In 2021, although the majority of foodborne outbreaks caused by toxigenic bacteria were related to *Bacillus cereus* toxins, the highest number of deaths and hospitalizations were mainly caused by *C. perfringens* and *S. aureus* toxins, respectively. The European Centre for Disease Prevention and Control (ECDC) has reported several outbreaks associated with contamination of different foods with these bacterial pathogens and toxins, emphasizing the importance of addressing contamination in the food supply [2].

Efforts to mitigate the microbial burden in food, particularly in raw products like fresh fruits and vegetables, involve an array of decontamination strategies, including thermal and non-thermal methods. However, these methods face inherent limitations [3,4,5]. Therefore, there is a pressing need for more efficient, safer, and environmentally friendly approaches to reduce food contamination.

Bacteriophages are increasingly recognized as biocontrol agents with enormous potential for the food industry. Bacteriophages, or simply phages, are viruses that specifically infect bacteria, the most abundant entities on the planet. Phages are obligate intracellular parasites that can reproduce only in the presence of a host bacterial cell. These viruses are innocuous to humans, animals, and plants [6], making them a safe alternative to conventional antibiotics and other treatments used in the food industry [7].

The interest in phages is clearly highlighted by several phage-based products for direct application in food, with Generally Recognized as Safe (GRAS) status, to control some of the leading foodborne bacterial pathogens that have already reached the market. Examples of commercial phage companies with Food and Drug Administration (FDA) approval for their food safety products include Micreos Food Safety, Intralytix, FINK TEC GmbH, Phagelux, and Passport Food Safety Solutions [8,9].

Several reviews have appraised the application of approved phages and also of other new phage suspensions directly in food against some of the most important foodborne pathogens [8,10,11,12,13,14] or specific bacteria, namely *E. coli* [15], *Salmonella* spp. [16], *L. monocytogenes* [17], and *Campylobacter* spp. [18]. Also, some review papers focus on the direct application of phages in a specific type of food, specifically on poultry meat against *Salmonella* spp. [16,19,20,21]. However, the successful application of phages in food still requires strategies to ensure their stability during food processing, transportation, and storage [9].

Many food environments subject phages to challenging physicochemical conditions, such as low pH levels on the surface of certain fruits and high storage temperatures during food transportation, which may lead to phage inactivation. The incorporation of phages into different materials are often employed in the context of active packaging systems designed to safeguard phages, maintain their stability, and facilitate controlled release, as recently reviewed by García-Anaya et al. (2023) [22]. In particular, this review addressed studies on phage incorporation in edible films and coatings and the associated challenges of applying them to foods.

The reviews available in the literature generally either discuss free phages or incorporated phages, some focus only on a specific bacteria or food, while others bring together pre- and post-harvest phage applications, with an integrative and detailed overview of phages for post-harvest food decontamination still missing in the literature. Thus, with this review, we attempted to fill this gap and gather here all the important information regarding food decontamination only at post-harvest level, focusing on the application of phages in their free form directly in food or incorporated into food packaging in a variety of different products and against the most important foodborne bacteria. This review examines some of the studies already outlined in other reviews but also includes the most recent articles in this field; since there is a growing interest in phages, this is an area in constant development and research. Regarding phage incorporation in food packaging, we tried to include all available relevant studies in the literature, considering that the majority of reviews only present a few studies on this topic.

**Figure 1:**
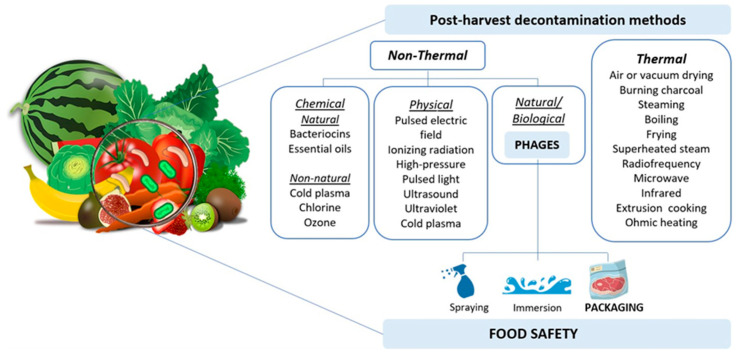
Post-harvest decontamination methods with a focus on phage application directly in food and phage incorporation into active food packaging.

In this context, this study offers an overview of available methods to reduce microbial contamination in food, with a particular focus on recent developments in the application of phages (Figure 1).

Specifically, we delve into the advantages and challenges of phages in the food industry, including their direct post-harvest application in food, and examine in detail some of the most recent studies on this topic. This review also includes the available *in vitro* and in-food studies involving the incorporation of phages into active food packaging systems.

## 2. Available Methods to Reduce the Microbial Burden in Food

### 2.1. Thermal Methods

Heat processing is one of the most efficient strategies used in the food industry to improve the quality and prolong the shelf life of food and includes pasteurization, sterilization, convective hot air drying, vacuum drying, burning charcoal treatment, steaming, boiling, and frying [23,24,25]. In these methods, the heat energy is transferred into a product through conduction or convection.

Pasteurization and sterilization are commonly used conventional techniques for the inactivation of enzymes and microorganisms in foods [26]. The difference between these two methods is the induction temperature. While pasteurization is performed at a temperature in the range of 60–80 °C to eliminate pathogenic microorganisms from food products and degrade enzymes, sterilization works at a temperature above 100 °C to destroy spores or spore-forming microorganisms in food products [27]. Pasteurization is commonly applied at a specific temperature and for a specific period of time in order to decontaminate and preserve liquid foods (milk, fruit juices, beer, and other fermented drinks) and pre-packed food products due to alarming food safety concerns related to ready-to-eat (RTE) food [26]. However, pasteurization does not completely eliminate all pathogenic microorganisms or kill heat-resistant spore-forming bacteria. Thus, moderate heat treatments between 40 and 60 °C are preferable to their high-temperature counterparts to reduce quality loss in food [23,25].

Superheated steam has higher heat transfer coefficients, which reduce microorganisms on the surface of food products efficiently with low energy consumption levels, non-oxidative conditions, and low environmental impacts. Applied for several food products, such as vegetables, fruits, cereals, and meat, this approach can facilitate the development of products with the desired quality, reduce nutrient loss, and improve the physicochemical properties of foodstuffs, solving the problems faced by traditional thermal procedures. However, this approach requires high maintenance costs due to the complexity of the equipment [24,28].

Infrared heating uses electromagnetic waves to transfer energy from the infrared source to the product. The heat needed for microbial decontamination is generated by the rotational and vibrational state of molecules or atoms [29] and also from hot sources like a metal rod, quartz tube, or quartz lamp. The germicidal property of this technology has been reported in different types of foods [30,31], namely in fruits [32,33], shelled corn [34,35], eggs [36], cottage cheese [37], and milk [38]. However, in food products with a complex matrix, radiation cannot reach the inner part of the sample and food heating is not uniform [39]. The food processing sector also uses infrared heating for blanching, thawing, broiling, frying, roasting, baking, drying, dehydration, peeling, polyphenols and antioxidants recovery, sterilization of grains, baking bread, manufacture of juices, and cooking food [30,31].

Table 1 summarizes the advantages and disadvantages of these and other post-harvest food decontamination techniques, which are further discussed in the following paragraphs.

Ohmic heating is based on the movement of an alternating electric current through a food matrix that is transformed into heat due to the electrical resistance of the food, allowing faster food heating when compared with conventional heating techniques. This process inactivates microorganisms mainly by thermal effects although other nonthermal inactivation mechanisms are also involved [40] and has been proposed as an alternative fast-heating method for fruit juices [56,57]. This method can also be effective for viscous products and pumpable food containing particles inside the food matrix due to the heat generation. Nevertheless, it requires uniform conductivity to avoid cold spots (Table 1) [40,58].

Microwave and radio-frequency heating treatments involve high-frequency alternating radio waves, microwave electromagnetic radiation, or electric fields [23].

Microwave treatment is applied in the food industry for thawing, blanching, precooking, cooking, tempering, baking, puffing, foaming, concentration, drying, pasteurization, and sterilization. The inactivation of microorganisms is caused by the denaturation of cell protein structure, leading to the extrusion of cellular matrix and death [29]. This technology has been studied in liquid foods, like grape juice, apple juice, apple cider, coconut water, and milk [23]. However, at high power levels, microwave heating can cause acrylamide formation (a neurotoxic and carcinogenic substance), unlike conventional food heat treatments. On the other hand, short exposure to microwaves (during blanching and thawing) at low power may even limit the formation of acrylamide during the final heat treatment (Table 1) [59].

Radio-frequency heating has been already used in the food industry for a considerable period post the baking of biscuits and cereals as well as the drying of food. Deeper penetration, compared to microwave treatment, makes this process more efficient. The heat causes inactivation and changes in the cell membrane of microorganisms and has been used to decontaminate different types of foods (meat products, salmon caviar, eggs, pasta products, peanut butter, crackers, sandwiches, fresh carrots, fruit juices, apple cider, whole milk, and soybean milk) [60]. Its main limitation is the inconsistent heating of the food matrix, which can impact food quality and safety (Table 1) [23,40,60].

Extrusion cooking combines heat transfer, mass transfer, pressure changes, and shear to produce different effects like cooking, kneading, shearing, shaping, and forming, among others [41]. This technique has gained popularity in the food and feed industries due to its low cost, high productivity, and enhanced product quality by retaining the heat-sensitive components of food (Table 1) since high temperatures are applied for short periods [41]. The extruded products have less moisture content, longer shelf life, and are considered microbiologically safe due to enzyme inactivation and microbial reduction. The success of extrusion cooking has been mainly reported in breakfast cereals, coextruded snacks, texturized vegetable proteins, and pet food; however, despite the advantages, this approach can cause changes in the physical properties of the treated food products [4,61].

### 2.2. Non-Thermal Methods

#### 2.2.1. Chemical Methods

##### Non-Natural Approaches

Washing, sanitation, and use of oxidative and non-oxidative biocides are commonly employed for the disinfection of food. Washing is responsible for removing oil, dirt, physical matter, and debris but also helps to reduce the microbial populations on the surface of vegetables. The addition of sanitizers to the washing water can increase the efficiency of disinfection, mainly in leafy vegetables. The majority of common sanitizers are oxidizing agents and work by developing oxidation potential in water [46]. Several sanitizing agents or disinfectants (including chlorine-based products, hydrogen peroxide, organic acids, and ozone) have been approved in order to reduce bacterial populations on minimally processed food, such as fruits and vegetables [5,42].

Chlorine is a commonly used disinfectant in the fresh produce industry. However, studies indicate that chlorine concentrations traditionally used are not effective in reducing pathogen loads on fresh-cut produce [42]. In addition, the use of chlorine as a sanitizer, as well as the use of other chemical compounds for food decontamination, might result in the formation of undesirable by-products such as carcinogenic compounds (Table 1) [62], with their use forbidden in several countries, namely European countries like Belgium, Switzerland, and the Netherlands [46,63].

Ozone is used for the surface decontamination of fruits and vegetables, drinking water disinfection, meat, and seafood processing. This sanitizer is effective against a broad spectrum of microorganisms, including bacterial spores [64], by interfering with their cellular respiration and interacting with the unsaturated lipids of the cell membrane causing microbial death However, ozone is not suitable for the treatment of liquid food and products rich in unsaturated fats and soluble proteins, and, as a powerful oxidant, must be handled carefully to ensure the safety of employees and durability of equipment (Table 1) [3,64].

Cold (non-thermal) plasma is one of the more recent and promising food decontamination technologies. This method uses a partial ionized gas composed of positively and negatively charged ions, electrons and neutral atoms, molecules, and radicals to inactivate microorganisms in food and packaging materials, namely by membrane rupture, oxidation of proteins, and nucleic acids damage. Due to the low penetration levels, the inactivation of microorganisms only occurs on the surface of solid food (Table 1) [48]. However, Xiang et al. (2022) reported the efficiency of water and other plasma-activated liquids in disinfecting food products with complex matrices [65]. This method is suitable for the surface treatment of fresh meats and greens and for seafood preservation [3,40,48,65].

##### Natural Approaches

Lactic acid bacteria produce significant metabolites from which bacteriocins, antimicrobial peptides, are particularly important because of the applications that these peptides may have in the food area. Bacteriocins act mainly by forming pores in the cell membrane of bacteria, causing apoptosis [66,67]. Since nisin received GRAS status from FDA in 1988 [68], numerous bacteriocins have been discovered and reported on; some of these are also already commercially available [69,70]. Bacteriocins are used to preserve meat and dairy products and are also applied as a bioprotective technology in fruits and vegetables [44,66,71,72] to reduce the cell counts of problematic strains, such as *Salmonella enterica* subsp. *enterica* serovar Typhimurium, *E. coli*, and *L. monocytogenes* [42,66,73]. However, some challenges (Table 1), namely those associated with their limited range of activity, can impair their efficacy [42,43,44].

Essential oils are widely used as flavorings for food and beverages, but several articles have demonstrated their capability as preservatives in different types of food due to their ability to inhibit the growth of or to eliminate pathogens [45,74,75] such as *Salmonella* spp. [76,77,78], *E. coli* [76,77,79], and *L. monocytogenes* [76,77,80]. Salanță and Cropotova (2022) recently reviewed the current knowledge on the applicability of essential oils in food preservation, reporting that the levels of inactivation differ between the used oils [75]. Also, in some circumstances, to achieve the same *in vitro* inhibitory effect, a higher concentration is needed to treat fruits, leading to possible negative effects on sensory properties that may limit their application (Table 1) [74].

#### 2.2.2. Physical Methods

Pulsed electric field is an environmentally friendly and highly energy-efficient technique that uses high-intensity short electrical pulses for food preservation with minimal loss in food quality and operates by two fundamental processes, namely electroporation and electrical breakdown [81]. This approach has been mostly applied for the treatment of orange, tomato, apple, and carrot juices [81]; apple sauce, pea soup; salad dressing; eggs [82]; and milk and milk products [83]. This technique increases the durability and safety of the products, retaining their sensory and nutritional characteristics. However, studies at the industrial scale are still very scarce [84]. The high start-up costs are one of the main barriers to the application of pulsed electric field on a large scale (Table 1) [40].

High-pressure processing is based on the treatment of liquid or solid foods at extreme pressures that can alter the anatomy of the bacterial cells as well as their enzymatic functions, resulting in their weakening and eventual death [3,40]. This procedure has been used commercially for several years, and its use is increasing continuously; it is employed in the treatment of foods whose water activity exceeds 0.8, such as fruits, meats, vegetables, milk and milk products, juices, beverages, seafood, and fish [3,40]. Despite the efficiency of this technology, and similarly to the pulsed electric field method, high-pressure processing also requires high initial costs associated with the establishment of appropriate facilities and the acquisition of the equipment (Table 1) [3,40]. In addition, this approach can influence sensory properties of foods [85].

Ultrasound is a safe, environmentally friendly, simple, and economically cheap (Table 1) technology that involves pressure waves with a frequency range of between 20 and 100 kHz, classified as minimum- or maximum-intensity sonication or, according to their frequency ranges, as power, high-frequency, or diagnostic ultrasound methods [86]. Many studies have demonstrated the potential of ultrasounds in eliminating microorganisms from meat, fruits, vegetables, dairy products, and eggs, with the preservation of physicochemical, bioactive, and nutritional components of the foods [3,40,46,49,86,87,88,89,90]. However, this technology is associated with negative effects on food characteristics, such as sensory parameters and nutrient composition (Table 1) [3,40,46,49].

Ultraviolet (UV) radiation is a non-ionizing radiation with germicidal properties at wavelengths in the range of 200–280 nm (UV-C). UV light can directly affect microbial pathogens by modifying their genetic material and/or damaging their proteins/lipids. Usually, different types of food products require specific doses of radiation to inactivate different types of pathogens. Apart from its lethality against all types of microorganisms, UV light also possesses other advantages, such as leaving no chemical residues, being easy to set up, and using low amounts of energy. Still, it also has certain drawbacks, for instance, it discolors some foods, only acts on the food’s surface, and exposure is harmful to humans (Table 1) [91]. Several studies have examined the application of UV-C light in the food industry for the surface decontamination of fresh foods, increasing the shelf life of foods, the pasteurization of fruit juices, and drinking water disinfection [3,23,46,50].

Pulsed light rapidly inactivates pathogenic and food spoilage microorganisms through the application of a comprehensive array of short but highly energized pulses; the wide band of white light affects microbial structures and leads to their inactivation [92]. The potential of this method has already been reported for a variety of foods [51], such as fish [93], meat [94], fruits [95,96], vegetables [96,97], milk [98], fruit juices [92], and water [99]. Pulsed light has been shown as suitable for application in liquid and regularly shaped foods due to its low degree of penetration. Also, long treatment periods can result in a ‘heating effect’ in food products that can impact the effectiveness of microbial destruction (Table 1) [3,92].

Ionizing radiation involves the application of three major types of ionizing radiation: (i) gamma, (ii) electron beam, and (iii) X-rays. This technology inhibits the enzymatic activity of microorganisms through direct acid nucleic damage and the production of reactive molecules that can lead to metabolic pathway damage inside the cells, intracellular oxidation, and consequently, cell lysis [3,40,52,100]. Several studies showed that this approach is effective in eliminating microorganisms in fruits, vegetables [3,40], and fish and meat products [101], thus preserving the physicochemical properties of the foods (Table 1). However, in some cases, in order to achieve a substantial level of microorganism elimination, a higher dose of irradiation is required, leading to food deterioration and risking processors and workers [63]. The huge capital/investment requirements associated with the purchase of an ionizing radiation facility and concerns about consumer acceptance are further disadvantages of this technology (Table 1) [3].

#### 2.2.3. Biological Methods

Bio-preservatives may originate from microorganisms, plants, and animals, being a potential alternative to the physical and chemical processing of foods [102]. Although bacteriocins and essential oils are considered natural chemical methods, as previously mentioned, they are also considered biological methods. Lactic acid bacteria, yeast, phages, bacteriocins, and endolysins are among the most common bio-preservatives effective in eliminating pathogens and preventing food spoilage [103]. These can be applied to food systems as additives, direct ingredients, or protective cultures. For instance, bacteriocins help in food bio-preservation, acting as antagonists, inhibitors, and antimicrobials against pathogens and spoilage microorganisms. In addition, they also provide various health benefits to humans, reinforcing the human immune system [104]. Yeasts are also known for their positive role in food fermentation, extending the shelf life of foods by creating an hostile environment to spoilage microorganisms [105].

Phages can be used as an eco-friendly approach to prevent and control pathogenic bacteria in food, with a substantial number of research reports describing the use of phages in various types of foods, ranging from meats [106,107,108] to fresh produce such as fruits [109,110] and vegetables [108,109,110], with promising results. Information regarding phages will be discussed in more detail in the following section (Section 3).

In addition to all the aforementioned food decontamination approaches, there is also the possibility of combining some of them, namely thermal and non-thermal methods, in order to take advantage of the benefits of each, resulting in a synergistic effect. Sometimes, these combinations can achieve more significant reductions in microorganisms than the individual treatments alone while retaining product quality. Information regarding these combinations can be seen in detail in different reviews [4,6,23,46,47,72,111].

## 3. Phages Against Foodborne Bacterial Pathogens

Phages were identified at the beginning of the twentieth century; however, a poor understanding of the nature of phage–host interactions and mechanisms underlying bacterial pathogenesis led to a succession of badly designed and executed experiments. Despite this, their potential as therapeutic antibacterial agents has been recognized. Phages were used to treat and prevent bacterial infectious diseases both in Eastern Europe and the former Soviet Union, but in the 1940s, with the advent of antibiotics, the application of phages as antibacterial agents diminished in much of the world [112]. However, the use of phage therapy has continued in regions of the former Soviet Union. Lately, with the emergence of pathogenic bacteria resistant to antibiotics, there has been a renewed interest in the use of phages as an alternative to conventional antimicrobials [12,54,113].

The differences in the infection process depend on the phage life cycle, which can be either lytic or lysogenic. In the cases of the lytic cycle (lytic phages), after the attachment and injection of phage genome into the host cell, the host DNA replication and protein synthesis machinery is exploited to produce new phage particles, which are released from the cell, culminating in the death of the bacterium. In the case of lysogenic cycle, the phages (designed as temperate phages) do not automatically start a lytic cycle, but instead the phage genome is integrated into the host cell genome (as a prophage) and may prevent the subsequent phage infection of the same host, thus delaying bacteriolysis. The induction, or excision of the prophage from the genome can occur spontaneously or in response to cellular internal or external triggers [114]. This results in the lysis and release of progeny phages that may progress and lyse or lysogenize other susceptible bacteria. The integration of the phage genome into the host genome may increase the virulence of the host. Temperate phages, in addition, may not cause the immediate death of the bacteria, but can be even favorable to bacterial evolution, particularly in regard to their pathogenicity and fitness. Through integration into the host genome, temperate phages can introduce new functional genes to the bacteria, such as virulence genes and antibiotic resistance genes, as reviewed by Chen et al. (2022) [115]. For these reasons, temperate phages should not be used in phage treatment unless they are genetically engineered.

Phage treatment uses lytic phages to destroy the host through a lytic life cycle [54,116,117,118].

Phages possess several benefits (Table 1) when compared to other post-harvest food treatment techniques that justify their use, including the following: (i) high specificity within a single genus or species or even within a subgroup of bacterial strains within a species, as opposed to some decontamination methods that indiscriminately kill bacteria that are naturally present and beneficial in food [119,120]; (ii) self-replication, which means that low or unique doses are enough for phage treatment as long as there are enough host cells to allow the phages to multiply [121]; (iii) no food safety issues related to the oral ingestion of phages in food, as phages are naturally present in high numbers in the environment and exist naturally on many food products and are thus often unintentionally ingested by humans [122]; (iv) phages do not affect the organoleptic properties, such as the sensory and quality characteristics, of food, unlike other post-harvest techniques such as thermal methods [12,122,123]; (v) self-limiting, since phages (in addition to being harmless) cannot persist for a long time in the environment without a host [124], unlike chemical compounds that have the capacity to leach into food produce and persist in their surroundings with a further risk of bacterial resistance; (vi) the purification, identification, and propagation of phages is relatively fast, simple, and production costs are considerably less expensive in comparison to other post-harvest decontamination methods [125,126,127]; and (vii) phages also have the ability to penetrate bacterial biofilms and infect cells contained within, which is unlike other decontamination methods that work effectively on planktonic cells [11,53,54,55].

Beyond the advantages, and like any other decontamination methods, phages also possess some disadvantages (Table 1): (i) phages may not completely kill or inhibit the pathogen and bacteria can naturally develop resistance to phages [128]; (ii) phages, mainly temperate phages, can transfer genetic material by horizontal gene transfer, which may increase the virulence of the host bacteria [129] and their antibiotic resistance [130]; (iii) phages are often exposed to, and inactivated by, certain factors like UV, pH, temperature, and osmotic pressure during different stages of food production, transportation, and storage [119,131,132,133,134,135]; (iv) the food matrix and the composition of the food surface (including food additives) can affect the efficacy of phages both by limiting phage diffusion or by specifically interfering with phages, thus reducing their encounter with host bacteria [123,136,137]; (v) release of pro-inflammatory compounds including endotoxins from lysed bacterial pathogens [121]; (vi) it is necessary to know which pathogen(s) are present to ensure that the correct phage or phage cocktail is applied [138]; (vii) the components of the phage product must have a host range broad enough to kill all members within the target pathogenic genus, species, or strains [9,11]; and (viii) in order to work, phages need to be applied in high enough quantities that allow phage particles to physically encounter all or most of the target bacterial cells, i.e., the initial phage titer applied to food samples needs to be high enough, and this is a determinant variable for the success of phage treatment [9,11].

Regarding the problem of phage-resistant bacteria, the combination of multiple phages (phage cocktails) [139,140] or the combination of phages with other antibacterial strategies in a multi-hurdle approach [141,142,143] may help to deal with the development of phage resistant-bacteria and thus inhibit pathogens, ensuring food safety. Komora and colleagues reported the synergistic effect of a multi-hurdle approach, combining mild high-pressure processing, phages, and bacteriocin against *L. monocytogenes* in milk [141] and in a fermented meat sausage model [142]. These two types of food matrices seem to be protective matrices that support phage application with mild high-pressure processing [143]. Promising results were also obtained with phages combined with essential oils and UV light against *L. monocytogenes* in beef, resulting in higher bacterial inactivation relative to each treatment individually [144].

Despite these strategies, bacterial resistance to phages is not as challenging as resistance towards antibiotics, since bacteria and phages co-evolve in the natural world, with phages evolving and adapting themselves to overcome bacterial resistance mechanisms [145]. Moreover, phage-resistant bacteria frequently exhibit a reduction in their fitness. Phage resistance frequently triggers several trade-offs in pathogenic bacteria, including a decrease in pathogenicity, a delay in bacterial growth, and even an increase in antibiotic susceptibility [115]. Phage engineering to increase bacterial host range is another strategy to deal with the emergence of phage-resistant bacteria [146].

Due to horizontal gene transfer, temperate phages must be avoided, and lytic phages must be characterized in terms of antimicrobial resistance, virulence, or integrase-coding genes to ensure their safety [9]. Despite these challenges, phage biocontrol is increasingly recognized as an attractive method to eliminate spoilage and pathogenic bacteria from food [42,117,122].

### 3.1. Use of Phages in the Food Industry at the Post-Harvest Stage

**Figure 2:**
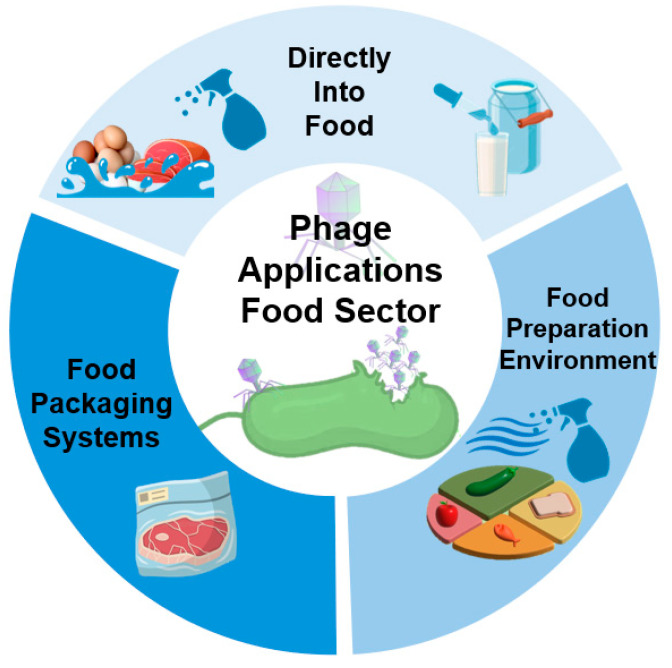
Phage applications in the food sector, highlighting the applications addressed in this review: direct phage application in food and their incorporation into food packaging systems.

Since the regulatory acceptance of ListShield™ in 2006, the first phage-based product approved by the FDA for use in the control of *Listeria* in meat and poultry products, the number of new available phage-based products for foodborne pathogen control at the post-harvest stage has increased [147]. Phages can be used in the post-harvest stage directly into food, food processing equipment surfaces and incorporated into food packaging systems to control food contamination [9,147]; in this review, only the use of free phages directly in food or incorporated into packaging systems will be addressed (Figure 2).

#### 3.1.1. Approved Phages for Direct Post-Harvest Applications in Food

Some private companies have demonstrated their intention to work on phage-based solutions to control or prevent foodborne pathogens in the food industry, but only a few products have been developed and are available in the market for direct post-harvest applications in food. (i) Micreos Food Safety (Wageningen, The Netherlands) developed a phage-based application to fight *L. monocytogenes* (PhageGuard Listex^TM^) in RTE meat products; fish, fruits, vegetables, cheese, seafood, and pet food; *Salmonella* spp. (PhageGuard S^TM^) in pork and poultry products, fruits, vegetables, and pet food; and *E. coli* 0157 (PhageGuard E^TM^) in beef carcasses, subprimals, beef cuts, and trimmings intended for ground beef, poultry, fruits, vegetables, and pet food. (ii) Intralytix Inc. (Baltimore, MD, USA) has commercialize phage-based products for controlling *L. monocytogenes* (ListShield^TM^) in RTE meat, fish (also smoked fish), and fresh and processed fruits and vegetables; *Salmonella enterica* (SalmoFresh^TM^) in poultry, red meat, fish, shellfish, fresh and processed fruits, and vegetables; *Shigella* spp. (ShigaShield^TM^) in cooked beef and chicken, smoked salmon, honeydew melon, lettuce, and yogurt; *E. coli* (EcoShield PX^TM^) in meat, poultry, fruits, vegetables, dairy products, fish, and other seafood; *E. coli* O157:H7 (EcoShield^TM^) in red meat parts and trim intended to be ground; and *Campylobacter* spp. (CampyShield^TM^) in raw red meat (including whole carcasses, primals, subprimals, cuts, trimmings, and organs) and raw poultry. (iii) Phagelux Inc. (Montréal, QC, Canada) supply a phage cocktail called SalmoPro^®^ for the biocontrol of *Salmonella enterica* on poultry, red meat, fresh and processed fruits, fresh and processed vegetables, eggs, fish and shellfish. (iv) FINK TEC GmbH (Hamm, Germany) developed a phage-based product called Secure Shield E1 for control of *E. coli* in beef carcasses [8,9,147,148].

#### 3.1.2. Recent Studies on Direct Post-Harvest Phage Treatment in Food

Recent years have witnessed successful phage-based biocontrol measures against some of the most problematic foodborne bacteria. Some of the most recent studies on phage application (mainly by immersion, pipetting, and spraying) against *Salmonella* spp., *E. coli*, *L. monocytogenes*, and *Campylobacter* spp. using approved or new phages in different foods are discussed in the following sections.

##### Phages for Biocontrol of *Salmonella enterica*

*Salmonella* spp. are a common cause of foodborne disease outbreaks and are recognized as one of the top four global causes of diarrheal diseases [149]. Salmonellosis is the second most common zoonotic disease after campylobacteriosis in the European Union [2]. *Salmonella* colonizes a broad range of animals and can then be transmitted to humans through eating, in particular, through contaminated animal-based food. *Salmonella* species are most frequently found in meat, dairy products, and eggs [150,151] but also can also be detected in fruits, vegetables and cereals due to cross-contamination during harvest and post-harvest periods [152].

Of the most recent studies on the post-harvest application of phages in food, those concerning *Salmonella* spp., namely *Salmonella enterica* subsp. *enterica* serovar Enteritidis [153,154,155,156,157] and *S.* Typhimurium [154,158,159,160,161], are the most common.

Meat [153,154,158,160,162], vegetables [159,163,164,165], dairy products [153,163,165,166], and eggs [155,156,164,167] are the most discussed food products. Other food matrices, such as fruit or vegetable juices [153,168,169,170], fish [171,172], seafood [172,173], water [153,174], fruits [175,176], and beef broth [169] were also reported on, but in smaller numbers. In these studies, new or previous isolated phages were mostly applied as single phage treatments [153,155,158,159,166,177]. A smaller but still significant number of studies also examined the treatment of food products with phage cocktails [154,156,157,161,162,167]. A few studies also investigated commercially approved phage cocktails, including SalmoFresh^TM^ [178] and PhageGuard S^TM^ [179,180,181].

Recent studies reported a significant *Salmonella* reduction in different food matrices after phage application through pipetting [171], immersion [177], and spraying [154] with bacterial reductions ranging from 1.7 to 4.5 log CFU, depending on the incubation temperature, MOI, and food matrix. Information regarding the detailed results of these studies can be found in Supplementary Table S1.

##### Phages for the Biocontrol of *Escherichia coli*

*E. coli* is responsible for severe foodborne infectious diseases. In foodborne *E. coli*, STEC strains are the most reported in outbreaks, accounting for 20% of global foodborne illnesses [182]. Enterohemorrhagic *E. coli* (EHEC) strains, especially *E. coli* O157:H7 (a STEC strain), are major pathogens associated with a higher risk of severe bloody diarrhea and hemolytic uremic syndrome when compared with other pathotypes of *E. coli* [183]. Meat and dairy products are among the foods most frequently contaminated with this pathogen [184,185]. Nevertheless, fruits and vegetables have also been implicated in *E. coli*–related illness [5,186,187,188].

Of the studies on recent phage treatments against *E. coli* directly in food, those concerned with the *E. coli* O157:H7 strain were the most frequent [189,190,191,192,193]. Also, the majority of these studies were performed on meat [189,191,194,195,196] and vegetables [189,192,194,197,198,199], followed by dairy products [189,194,195,197,200]. The potential of phages against *E. coli* on chicken skin [197,201], fish [109,202], eggs [201], fruits [109], seafood [173], and cooked rice [194] was only addressed in a limited way. Most studies applied new or previously isolated phages separately [189,192,194,197,198,200] or combined in phage cocktails [190,195,199,203], with a reduced number of studies using approved phage products, namely PhageGuard S^TM^ [191], PhageGuard E^TM^ [108,204], and EcoShield^TM^ [109,205].

Promising results on *E. coli* reduction in different types of food were obtained in recent studies after phage application by pipetting [189,206] and immersion [192]. In general, higher bacterial reduction was observed in liquid compared to solid matrices with reductions of 0.7 log CFU/piece in meat and 4.7 log CFU/mL in milk. For detailed results please consult Supplementary Table S1.

##### Phages for the Biocontrol of *Listeria monocytogenes*

*Listeria monocytogenes* is a foodborne pathogen responsible for causing listeriosis. Listeriosis is the third leading cause of death from foodborne illnesses, with about 1600 people suffering from listeriosis each year and about 260 dying [207]. Seafood, soft (raw milk) cheese, unpasteurized milk, and meat spreads are regarded as moderate- to high-risk foods for *L. monocytogenes* contamination in retail. Also, fresh or minimally processed fruits, such as apples and lemons, and vegetables are often associated with infection with this bacterium [208,209,210]. The ability of *L. monocytogenes* to replicate at refrigeration temperatures makes it one of the most important pathogens in RTE food [211,212].

Despite the reduced number of post-harvest studies using phages against *L. monocytogenes*, compared to *E. coli* and *Salmonella*, phage potential against this bacterium has been evaluated on different food matrices, including meat [142,213,214,215,216,217,218], vegetables [215,219,220,221,222,223], fish [224,225,226], dairy products [141,216,222,227,228], and fruit juice [226]. The majority of studies focus on the use of the approved phage product PhageGuard Listex^TM^, containing a single *Listeria* phage P100 [141,142,216,217,220,221,223,224,225]. The approved phage cocktail ListShield™ was also applied in a few studies [218,227]. Other works report the use of new or previously isolated single phages [222,226,228] or phage cocktail [214,215,219].

Recent studies reported a significant decrease in *L. monocytogenes* levels after phage addition, by pipetting [227] and immersion [215,219], to different foods. Despite the differences in the tested matrices, in general, a maximum reduction of around 2 log CFU/g was obtained. The detailed results of these studies can be found in Supplementary Table S1.

##### Phages for Biocontrol of *Campylobacter* spp.

*Campylobacter* spp. infections are the most common bacterial cause of human gastroenteritis in the world [229]. Within the *Campylobacter* genus, the species *Campylobacter jejuni* and *Campylobacter coli* are considered the most important human pathogens, involved in most cases of campylobacteriosis. Campylobacteriosis was the most reported zoonosis in the European Union in 2021 with poultry meat and milk as the main sources of *Campylobacter* infection in humans [2]. Several control strategies for *Campylobacter* spp. have been developed with most of them focused on the reduction in Campylobacter colonization at the farm level. Strict biosecurity measures, good manufacturing practice, hazard analysis and critical control points, vaccines, phages, bacteriocins, probiotics, and phytochemicals are among these strategies [18,230,231,232].

From the beginning of 2018 until now, there have only been two available studies on phage application in food which are concerned with the use of a new single phage against *C. jejuni* [233] and a previously isolated phage against *C. coli* [234], both at a post-harvest level on meat. The reduced number of *Campylobacter* studies is probably a result of the difficulty of working with *Campylobacter* phages [235].

Despite the reduced number of studies against *Campylobacter*, promising results were reported after phage application by spraying [233] and pipetting [234] in meat, with reductions between 1 and 2 log CFU/g. More detailed results can be found in Supplementary Table S1.

#### 3.1.3. Factors Affecting the Effectiveness of Phage Treatment Directly in Food

Despite the numerous studies and promising results, there are some challenges associated with phage application in food. One of the main limitations is phage vulnerability towards various conditions [236,237], and it is difficult to maintain a constant phage titer due to the complexity of some foods [236,237]. Whether applied as a prophylactic or as a disinfectant, phage stability is very variable and depends on certain parameters, such as phage type, food composition, temperature, pH, and the presence of antiphage compounds [238]. Phages are often exposed to, and inactivated by, extreme environmental factors and physicochemical conditions in the food matrices. Extreme pH (common in fruits and fruit juice) [133], high temperature [133,239,240], salinity [133,240], UV irradiation [132,240], desiccation [132], and antiphage food compounds, such as milk caseins and organic acids and tannins from fruits and vegetables [236,237], have all been demonstrated to significantly reduce the concentration of viable phages and their activity. These factors can lead to damage in structural protein elements of a phage (capsid, sheath, tail) as well as lipid loss and/or can promote DNA or RNA structural changes [241]; therefore, these must be considered during food processing, transportation, and storage in order to maintain phage viability and ensure the effectiveness of the treatment [9].

The protein structure of phage particles determines its stability [242]. Tailed phages are known as the most stable in adverse conditions, and phages with larger capsids have higher survivability [133,241]. Temperature and pH are the main factors limiting phage activity [238]. Low and high pH values and high temperatures tend to inactivate phages [133].

Temperature is a fundamental factor for phage stability and activity [240,243,244,245], playing an important role in the attachment, invasion, and replication of phages [133]. At low temperatures, only a small number of phage particles inject their genetic material into the host cell and, thus, only a few are involved in the phage amplification stage [246]. On the other hand, higher temperatures can lead to the denaturation of proteins from the phage capsid [238,247]. Phages exhibit optimum propagation at temperatures of 37–40 °C [248]. However, several studies confirm that thermal stability is specific to each phage, and it is different depending on the phage isolate. Vörös et al. (2018) showed that high temperatures reduced the infection of the host by phage T7 since the high temperature caused the tail of the phage capsid to break [249]. Other studies suggested that high temperatures enhanced infection since they provided more energy to drive the genome into the host cells [250,251,252]. Ahmadi et al. (2017) reported that phage A511 was more stable when infecting *L. monocytogenes* at high temperatures (60–80 °C) than the phage P100 due to the slightly higher protein melting point of the tail capsid connector protein of phage A511 [239]. Additionally, some phages are insensitive to temperature. For example, in a study from Taj et al. (2014), the myovirus phage T4 effectively lysed *E. coli* BL21 at temperatures between 15 and 41 °C [253]. Also, the phage P100 in *L. monocytogenes* remained infectious throughout a range of 4–60 °C [131,239].

Regarding pH, in general, most phages are stable within a pH range of 4–11 [254] and their optimum pH conditions are around a neutral pH of 6–8 [133]. Several studies have suggested that the major limitation for the use of phages as natural preservative in fruits is the acidity of the environment (pH < 4) [143,255,256,257]. Oliveira et al. (2014) tested phage P100 stability on melon, pear, and apple products (juices and slices). Phage P100 was stable at 10 °C for 8 days in melons and pears (pH 4.61 to 5.92), but 2 and 7 log PFU reductions were observed in apple juice and apple slices (pH 3.70 to 3.76), respectively [257]. Also, Komora et al. (2018) showed a lower resistance to the pressure of phage P100 for lowest pH values. An accentuated phage titer reduction in phage P100 was observed in apple juice (pH 3.41) and orange/carrot nectar (pH 3.54) treated with high pressure, which may be related to the acidic pH values of these matrices compared to the other tested matrices, namely UHT whole milk (pH 6.73), “Serra da Estrela” cheese (pH 5.66), and “Alheira”, a meat sausage (pH 6.07), which supported phage P100 application in high-pressure processing up to 300 Mpa [143].

Like any other viruses, most phages are also susceptible to UV radiation, which is responsible for intriguing adsorption flaws [258]. Since lethal UV radiation photoproducts are normally thymine dimers, DNA phages are usually more sensitive to UV damage than RNA phages [243] and, generally, phages with double-stranded genomes are more resistant to UV radiation than single-stranded ones [259,260,261]. UV radiation is more problematic in certain applications, such as crop treatment or aquaculture infection control, where phage inactivation by UV light significantly limits phage efficacy [124,240,262]. However, when directly treating foods at the post-harvest stage, food must also be protected from UV radiation to ensure the effectiveness of the treatment. In a study by J. Zhang et al. (2015), the direct exposure to UV light (302 and 365 nm) significantly reduced the concentration of ten Salmonella phages after 30 s, with no detectable viable phages after 900 s of UV light exposure (302 or 365 nm) [263]. In another study, Hudson et al. (2016) [264] reported the inactivation of *E. coli* O157:H7 NZRM 3614 by untreated and UV-treated phage FAHEc1 in milk and RTE meat. In milk, at 5 °C, a bacterial reduction of about 4.5 log by viable phages was observed after 35 days, whereas UV-treated phages led to a bacterial reduction of only about 1 log. In RTE meat, at 5 °C, higher bacterial reduction was obtained with viable phages (1.5 log) compared with the obtained reduction with UV-treated phages (0.5–1 log), after 24 h. At 24 °C, the reduction produced by the viable phages was once again higher (around 4.5 log) than that produced by UV-treated ones (3.5 log) after 24 h. The results confirmed the impact of UV on phage viability and, consequently, on bacterial reduction levels [264].

Some studies also reported the effect of desiccation on phage survival [132,265,266,267]. Iriarte et al. (2007) showed the *in vitro* effect of desiccation (at constant darkness, room temperature, and 0% relative humidity) on the survival of phage ΦXacm 2004-16 with reductions of 2.06 log PFU/mL after 60 days [132]. In another study, Rode et al. (2011) reported a 7 log reduction in phage Stx2 titer under desiccation on coupons of stainless steel at 20 °C [266]. More recently, Carrigy et al. (2019) observed phage CP30A titer reductions of 2.0 ± 0.1 log PFU/mL after air drying at room temperature for 48 h in a Petri dish [267].

In addition to environmental factors, other variables in particular the food matrix and the composition of the food surface can also affect the efficacy of phages [137]. The complex microstructures and matrices of food strongly affect the success of phage intervention, both by limiting their diffusion or by specifically interfering with phages, thus reducing their encounter with host bacteria [268]. In a study by Zinno et al. (2014), phage titers increased in all tested foods (whole milk, skimmed milk, energy drink, apple juice) except in liquid egg, in which a decrease probably occurred due to the highly viscous nature of the egg matrix, thus limiting diffusion and homogeneous phage distribution and leading to a decrease in phage numbers [269]. Similarly, in another study, Bao et al. (2015) tested a phage cocktail to reduce *S.* Enteritidis in milk, cabbage, and chicken breast and the highest effect was observed in milk, suggesting that the liquid allowed a greater diffusion of the phages [270]. This is reinforced by the lower phage titer and consequently lower MOI required for inactivation in liquids compared to solids, as observed by Thung et al. (2017), who used an MOI of 10^5^ to reduce *S.* Typhimurium load in liquid egg and fruit juice, while a higher MOI of 10^7^ was required to achieve a similar reduction in cooked beef and chicken [271].

Many food additives can also impair phage activity [143] with a consequent decrease in treatment effectiveness, namely dairy proteins, organic acids, fatty acids, tea infusions, phenolic compounds, retinoids, high ionic (NaCl), and sucrose concentrations [236,237,272,273,274]. Guenther et al. (2009) showed that lettuce and cabbage affected the stability of phage A511 by reducing the phage titer from 8 to 7 log PFU/g after 6 d at 6 °C, probably due to the presence of organic acids and tannins [268]. In another study, De Siqueira et al. (2006) found that tea infusions are also capable of reducing the viability of *S.* Typhimurium phages, Felix 01 and P22. Of the nine tea infusions tested, gold leaf tea had the highest antiphage activity with 7 to 8 log PFU/mL titer reductions for both phages [272]. García et al. (2009) also observed a phage titer decrease (about 1 log) throughout the incubation period in semi-skimmed and whole-fat raw milk assays. The authors suggested that fat globules and milk protein might promote phage particle aggregation and render them unable to infect the host under the assay conditions [273]. García-Anaya and colleagues (2019) described the interaction between phage A511 and the protein phases (whey and casein) in both unhomogenized and homogenized milk. The lowest phage affinity (0.06 to 0.48%) occurred in whey from both milk types. Nevertheless, in the casein phase, the affinity increased gradually with the degree of homogenization (from 8 to 20%). The authors proposed that this effect was associated with the exposure of both hydrophobic and hydrophilic sites in casein micelles during the homogenization, suggesting that phage–protein interactions and, consequently, phage inhibition can be modulated by alterations in milk proteins [275].

Different encapsulation strategies, such as extrusion, emulsification, and formation of liposomes, can help protect phages and maintain their viability in adverse environmental conditions, namely acidic environments and high temperatures, by the creation of phage microcapsules. Different polymers have been applied for phage encapsulation, with alginate as one of the most studied polymer materials in this context [276]. Several studies demonstrated the improvement in phage stability after encapsulation. The encapsulation of an *E. coli* phage (UFV-AREG1) in alginate, alginate–carrageenan, and alginate–whey protein spheres by extrusion was shown to increase its survival in simulated gastric fluid, especially in the alginate–whey–protein spheres [277]. Core–shell capsules encapsulating *E. coli* phage T3 by emulsification in a water-in-oil emulsion in the core with either alginate only or Eudragit S100 + alginate shell provided phage protection at pH 1 for 2 h. Also, encapsulated phages were significantly more stable than free phages upon exposure to temperatures up to 95 °C for 120 s [278]. *Salmonella* phages (UAB_Phi20, UAB_Phi78, and UAB_Phi87) encapsulated in liposomes were significantly more stable in simulated gastric fluid (pH 2.8), with losses of 3.7 to 5.4 log, compared to non-encapsulated phages that decreased by 5.7 to 7.8 log [279].

Another strategy of phage protection is its incorporation into active food packaging systems [8].

#### 3.1.4. Phages in Active Food Packaging Systems

The application of phages in their free form directly into food sometimes requires their preservation for longer periods of time in extreme conditions [280], such as those highlighted in the previous section. In most cases, uncontrolled release and fast activity and stability loss during storage may impact the effectiveness of phage treatment in food. Furthermore, the traditional methods of the application of free phages into food products (usually by immersion, pipetting, or spraying) [281] are often associated with dilution, requiring a high number/quantity of phages to be effective in order to suppress the bacterial growth [281,282].

In recent years, packaging, in addition to its main function of protecting food against external foreign environments and to increase ease of handling, has also started to play an active role in preventing food waste by actively extending its shelf life, thus ensuring quality and security [283]. Packaging systems can be classified into traditional or passive, active, intelligent, and smart [284]. The term “active packaging” is receiving significant attention, and it refers to packaging in which additional functions/ingredients have been purposely added to produce a more efficient packaging system, caring for the quality and safety of food and extending its shelf life, mainly by providing additional protection against the growth of microorganisms or the oxidation of a product [285].

The incorporation of phages as antimicrobial agents in active packaging systems could be explored in order to reduce the effects of external factors and food properties on phage stability and to promote the slower and continuous release of the phages into the food, enhancing food safety, extending food shelf life, and reducing phage waste that occurs with the traditional methods of phage application [8,281]. Moreover, it is expected that this approach will result in targeted and effective antibacterial actions due to phage specificity [11].

In addition, in order to minimize the environmental impact caused by traditional plastic packaging, fully biodegradable biopolymeric-based materials are gaining considerable relevance [286]. Polysaccharides and proteins are the most used biopolymers for food packaging development and production [287]. Water-soluble materials, such as some polysaccharides and proteins, are promising for phage incorporation, since, in general, the stability and viability of phages can be better maintained when they are stored in water-based solutions [288,289,290]. Plasticizers are commonly added to biopolymers in film-forming solutions in order to increase flexibility and improve the mechanical properties of films and coatings. Glycerol, polyethylene glycol, and sorbitol are among the most widely used plasticizers [291,292,293]. Glycerol can also act as a phage protectant [294].

Although both active coatings and films can control and prevent foodborne pathogens, these two terms have distinct meanings, as pointed out by García-Anaya et al. (2023a) [22]. Coating formulations are typically film-forming solutions that are applied directly to the food surface, usually by spraying, brushing, or dipping. After drying, they act as barriers that may be removed or consumed as a part of the food to provide protection, enhance the food’s appearance, or provide specific properties, such as antibacterial properties when containing phages or other active compounds. Films are thin materials obtained also from a film-forming solution after drying. These pre-formed films are often used as packaging materials to wrap the food or as coatings for other materials [22,295]. Several systems for phage incorporation and delivery, namely biopolymeric edible films and coatings, have been reported [22,295]; these aim to deliver phages to a specific site and allow them to remain intact and viable with prolonged activity and controlled release into food.

Phages can be incorporated into packaging films or coatings through different methods, with each possessing associated advantages and disadvantages [22,295]. The most common methods used to protect phages against adverse environmental and food processing conditions and to ensure their stability when incorporated into different materials rely on some form of encapsulation or simple immobilization into the packaging material. In the encapsulation approach, phages are encapsulated by certain stabilizing agents that confer protection against the external environment and added to a specific packaging material, requiring their release from the capsules in order to contact the target bacterial cells [280,290,296]. Immobilization is based on trapping phages within a matrix. Due to these differences, a small fraction of immobilized phages can be exposed to the external environment, which does not happen with encapsulation [280,297]. Despite the implications of the stability of immobilized phages, immobilization also allows for more direct contact between the phage and the target bacteria [280].

To the best of our knowledge, Table 2 outlines all available studies on phage incorporation into packaging materials, edible coatings, and coated packaging materials against different foodborne bacteria. Table 2 summarizes information regarding the target bacteria, the applied phage(s), the material(s) used for phage incorporation, the tested food products, and the major findings, namely the bacterial reduction, phage behavior, and the effect of phage addition on the properties of the films/coatings. In these studies, the most frequently investigated bacterium is *E. coli* [265,289,294,298,299,300,301,302,303,304,305,306,307,308,309]. Meat was the most tested type of food [265,289,300,301,302,307,310,311,312,313]. Among all the materials used for phage incorporation, cellulose derivatives [265,289,304,306,314,315], alginate [288,298,302,303,313,315,316] and whey protein [307,308,310,315,317,318] were the most frequently reported.

Although this review considers several studies targeting different bacteria, the focus of this review is on the most important foodborne pathogens, namely *E. coli*, *Salmonella* spp., *L. monocytogenes*, and *Campylobacter* spp., and the following subsections emphasize on the main findings on these specific bacteria. To date, there are no studies available in the literature regarding the incorporation of *Campylobacter* phages into food packaging systems.

##### Incorporated Phages Targeting *Escherichia coli*

Several *in vitro* [294,298,303,304] and food studies [265,299,300,301,302,305,306,307,309] with incorporated phages targeting *E. coli* have been reported (Table 2).

The majority of studies aimed to combat the serotype *E. coli* O157 [265,294,299,300,301,302,307] and most experiments were performed on meat [265,300,301,302,307], followed by fruits and vegetables [289,299,302,308,309]. Some studies were performed in milk [306] and in medium containing food components [305].

In most studies, a single phage, namely a T-even type phage, was used [300,303,304,305,306,308,309], and only a few studies reported the use of phage cocktails [265,289,307].

Cellulose and cellulose derivatives, including modified cellulose membranes, cellulose diacetate, and filter paper functionalized with carboxyl methyl cellulose or chitosan [265,289,294,304,306] were the most explored materials for the incorporation of the phages. Other studies included phage combination with whey protein [307,308,309], polyethylene oxide [302,303,304], chitosan [299,301], indium tin oxide [305], polycaprolactone [300], and sodium alginate [298]. Some of these studies reported improved phage stability with the addition of maltose and starch [265] and trehalose [294]. Improved antibacterial activity was also observed in the presence of D-phenylalanine [302]. Higher phage stability and, consequently, higher bacterial reduction were also observed when phages were first encapsulated before their incorporation into different materials [301,302,303]. The infectivity of the incorporated phages was maintained for 1 week [299,306], 4 weeks [294,308], and 5 weeks [307] with higher stabilities at refrigerated temperatures compared to room and higher temperatures.

Although refrigerated temperatures were better for maintaining phage stability, higher and/or faster bacterial reductions were observed at room and higher temperatures of 37 °C [300,307]. In the majority of the studies, higher bacterial reductions were obtained with free phages; however, in some cases, similar results between free and incorporated phages were observed [289]. In general, higher and faster bacterial reductions were obtained *in vitro* compared to in food assays.

##### Incorporated Phages Targeting *Salmonella* spp.

Regarding the incorporation of *Salmonella* phages into different systems (Table 2), both *in vitro* [294,298,314,319] and food studies [311,312,313,315,316,320] are also reported in the literature.

Studies targeting *S.* Enteritidis are the most frequent [298,311,313,319,320], but some examine other individual *Salmonella* strains [294,314,316] or mixtures of different *Salmonella* strains in bacterial cocktails [312,315].

Similarly to those on *E. coli*, most studies used a single phage [294,298,311,313,320], namely the commercially available phage Felix O1 [312,319], but also phage cocktails are investigated [314,315,316].

Food studies were performed on meat [311,312,313], fruit [315], cheese [316], and eggshells [320].

*Salmonella* phages were incorporated into coatings/films of chitosan [315], alginate [298,315,316], chitosan–alginate [313], cellulose derivatives [314,315], whey protein [315], polyvinyl alcohol [319,320], pullulan–trehalose [294], and poly(lactic acid) films coated with xanthan [312]. Also, phage incorporation into a hydrogel film was studied [311].

In a study with different polymers (whey protein concentrate, carboxymethyl cellulose, chitosan, and sodium alginate), films of whey protein concentrate showed the greatest results in terms of phage stability and antibacterial effects [315]. The addition of cinnamaldehyde to sodium alginate films also resulted in higher bacterial reduction; however, alterations in film properties were reported after the addition of this compound [298].

In some cases, phage addition did not result in changes to film properties [298]. However, in others [314], phage addition led to modifications in the film surface, reducing the transparency, tensile resistance, and modulus of elasticity of the film and increasing the porosity.

The stability of *Salmonella* phages in the different produced films/coatings varied from 14 days in cellulose acetate at room temperature [314] to 30 days in polyvinyl alcohol at 5 °C [320] and 60 days in pullulan–trehalose-coated paper at room temperature [294]. Similar antibacterial effects were observed with the layer-by-layer assembly of chitosan/alginate thin films that incorporated phages and free phages [313].

##### Incorporated Phages Targeting *Listeria monocytogenes*

Table 2 also includes several *in vitro* and food studies with incorporated phages targeting a specific *L. monocytogenes* bacterial strain [265,288,294,310,317,318,321] or a mixture of strains in a bacterial cocktail [289,312].

A majority of studies utilized phage A511 [310,312,317,318] and the commercially available phage LISTEX™ P100 [288,289,294,321]. Other studies also used phage cocktails [265,289].

From the available studies that utilized foods, meat was the most tested type [265,289,310,312]. However, there are also studies on fruit [289] and cheese [317].

*Listeria* phages were incorporated into whey protein [317] or into films composed of whey protein and pullulan [310,318], in positively charged cellulose membranes [265,289], xanthan coatings on poly(lactic acid) films [312], a pullulan–trehalose mixture [294,321], a pullulan–trehalose mixture coated onto packaging paper [294], sodium caseinate, and sodium alginate mixed with gelatine and polyvinyl alcohol [288].

Despite free phages in some cases showing a higher antibacterial effect than immobilized phages [288,289], in others, no significant differences in antibacterial effect between free and immobilized phages were observed [289]. Also, although some studies reported better results at refrigerated temperatures [288], in general, bacterial reduction was temperature-dependent with higher bacterial reductions obtained at room temperature [289] and at temperatures higher than 37 °C [312].

The stability of the incorporated *Listeria* phages varied from 16 days at 4 °C [317], to 20 days [310] and 60 days [294,318] at 25 °C.

Regarding film properties, some studies reported that phage addition altered film properties, such as opacity [318], while in others, the addition of phages did not cause changes in their properties [288].

In general, better results in terms of bacterial inactivation were obtained *in vitro* compared to the results obtained in food. Also, in food, the bacterial reductions were matrix-dependent. The lowest *L. monocytogenes* reduction was obtained in cheese with a long incubation period of 16 days at 4 °C [317].

Regarding phage stability, Leung et al. (2018) reported that drying method and humidity significantly affected the long-term viability of LISTEX P100 phages in pullulan/trehalose films, with trehalose also playing an important role in the enhancement of phage stability [321]. Also, other authors reported better results in terms of phage stability, bacterial reduction, and film properties in whey protein concentrate and pullulan blends instead of the corresponding pure films [310,318].

## 4. Summary and Future Perspectives

Phages in their free form or incorporated in active food packaging systems have proven to be an eco-friendly strategy to effectively decontaminate food and improve food safety. With several commercial phage products already approved and numerous studies demonstrating their antibacterial efficacy against problematic foodborne pathogens, phages hold great promise for future use in common food safety practices. However, there are significant regulatory, industrial, and implementation challenges that must be addressed to scale them up effectively.

Regulatory frameworks remain one of the key obstacles of phage use in the food industry. The approval process for phages as food additives, biocontrol agents, or components of packaging materials requires clear guidelines on their safety, efficacy, impact on food quality and environmental impact. There is a need for continued collaboration between scientists, food producers, and regulatory bodies to create a consistent regulatory framework that addresses specific concerns such as dosage, phage stability, and potential resistance development.

Industrial scalability also presents challenges, whether for direct phage application or incorporation into packaging. In both cases, ensuring the sustainable production of phages at a large scale is critical. Phage production for large-scale use in food safety applications needs to be both economically viable and able to meet the high-volume demands of the food industry. For active food packaging, phage incorporation must not only be cost-effective but also retain phage viability and efficacy under real-world conditions. For direct application to food, additional complexities include maintaining phage stability during transportation, storage, and application, especially when dealing with perishable items. Researchers are exploring methods such as encapsulation or the use of stabilizing agents (e.g., trehalose) to improve phage retention and activity, whether in packaging or in food applications.

Barriers to implementation are equally significant. Consumer acceptance remains a critical issue, especially given concerns about the safety of consuming phages, despite their natural occurrence in food. It is essential to educate both consumers and food producers about the safety, natural ubiquity, and efficacy of phages to overcome these barriers. This requires careful coordination between food producers, packaging companies, and regulators to create solutions that are both effective and practical at an industrial scale.

Further research to refine application techniques, ensure consistent performance across different food matrices, and evaluate the long-term impact of phage use is needed. For instance, understanding how phages interact with non-targeted commensal bacteria and ensuring that they do not disrupt either food microbiota or consumer microbiota or cause unwanted resistance in other microbial populations are important areas for future investigation. Phage combination with other decontamination methods, including application timings, also needs more research in order to maximize their effect and reproducibility.

## Figures and Tables

**Table 1:** Advantages and disadvantages of post-harvest food decontamination techniques.

Thermal Methods			
Type of Decontamination Method	Advantages	Disadvantages	References
Conventional thermal methods	Pasteurization, sterilization, air or vacuum drying, burning charcoal, steaming, boiling, frying Effective reduction in the microbial load in food products	High energy consumption High environmental footprints Poor food quality	[23,24,25]
Novel thermal methods	Superheated steam Fast processing rate High energy efficiency Enhanced safety Low environmental footprint Water saving Excellent product quality	Can lead to poor product quality High maintenance costs due to the complexity of the equipment	[4,24]
	Radiofrequency and microwave Cost-effectiveness Easy and quick operation High energy efficiency Non-toxicity Suitability for heat-sensitive fluids	Limited use to high-moisture, high-salt, and high-fat content food products Non-uniformity heating that creates hot and cold spots inside the food product affecting its microbial safety Microwave treatment can lead to moisture and nutrient loss	[4,23,40]
	Infrared Precise and rapid heating High heat-transfer efficiency and heating rate High levels of time and temperature control Easy operation Low maintenance costs	Relatively low penetrability Sometimes causes color degradation The equipment design and parameters must be optimized to avoid the overheating problem in industrial applications	[4,23,39]
	Extrusion cooking Low cost High productivity Enhanced product quality by retaining heat-sensitive components of food	Changes in the physical property of food products Puffing up of the food products	[4,41]
	Ohmic heating Rapid heating of the food Overcooking can be avoided Low heat losses No residual heat transfer after the current shut off Better impact on sensory properties of food Low maintenance costs Suitable for viscous products and pumpable foods containing particles	Requires uniform conductivity within the food matrix to avoid cold spots Might lead to considerable color differences	[40]
Non-thermal methods			
Chemical methods (natural)	Bacteriocins More stable and effective at: - Acidic pH - Higher-than-normal temperatures or lower-than-normal temperatures Degraded by proteases	Lactic acid bacteria have been linked with sepsis, endocarditis, and bacteremia May not be effective in satisfactorily reducing the microbial load Research must determine if the lactic acid bacteria strains have long-term effects on bacterial reduction during shelf life Limited range of activity	[42,43,44]
	Essential oils Natural origin Improve the flavor, odor, and color of food	The elimination of a specific microorganism in food is still limited Level required for inhibition may introduce too strong a flavor to the food	[42,45]
Chemical methods (non-natural)	Chlorine-based methods Inexpensive Easy to use	Generation of toxic by-products as well as off-tastes and odors May not be effective in satisfactorily reduce the microbial load on surfaces containing biofilm or in food matrices with high organic content	[42,46]
	Ozone Can be used in both a gaseous or liquid form Higher percentage of reduction in microorganisms compared to chlorine Relatively cheap with low running costs Environmentally sustainable and commercially feasible technology Short contact time for disinfection compared to other conventional methods	Oxidative spoilage of the food Limited to surface treatment High initial investment Unstable nature Highly toxic and corrosive compound with a pungent disagreeable odor Effectiveness diminished by the presence of organic matter	[3,42,47]
	Cold Plasma High percentage of reduction Broad spectrum approach Allows in package treatment Eco-friendly benefits: - diminished water utilization - absence of chemical residues - utilization of environmental air as a working gas Minimal changes in the food matrix	Low penetration efficiency High microbial loads reduce its efficiency Efficiency dependent on humidity, gas composition, and flow rate May result in lipid deterioration and the destruction of inherent antioxidants by the free radicals formed, leaving the food with an undesirable taste and aroma High capital investment Still at its lowest level (laboratory or pilot scale)	[3,48]
Physical methods	Pulsed electric field Retains nutrients and sensory characteristics, promotes durability, and ensures the safety of the foodstuffs Environmentally friendly and highly energy-efficient technique Decrease in energy costs, processing time and degradative effects of heat-sensitive food components	Huge start-up costs Some bacteria cells have developed resistance against this method Not yet effective in treating solid foodstuffs, compared to partial solids or liquid foods	[3,40,46,47]
	High-pressure processing Commercially available technique for bulk quantities of samples, either solid or liquid Retains the taste and nutrient composition of food and elongates its shelf-life Eco-friendly approach	Not suitable for dehydrated and porous foodstuffs The treated food must be kept in cold/refrigerated conditions Only plastic materials appear the best fit as packing materials High equipment costs Need for appropriate skill and space to effectively operate	[3,40,47]
	Ultrasound Commercially available technique for both solid and liquid food Highly reduced treatment time for handling food Uses a minimal amount of energy Safe and environmentally friendly technology Quite feasible, as it is simple and economically cheap Useful for eliminating microbial entities that can hinder the food fermentation processes	Free radicals may decline food product quality Harmful effects in food characteristics such as sensory parameters as well as nutrient composition Better reduction when applied in combination with other treatments The sonication is more confined to liquid foods	[3,40,46,47,49]
	Ultraviolet radiation Better nutrient preservation Higher lethal effects against microbes when compared to some conventional chemical agents Easy to use, cost-effective, and environmentally friendly approach Minimal effects on the quality of food Can be used for liquid and solid food Prevents recontamination as it can be applied in already packed food products Its processing time is described as shorter	Lack of complete recognition and acceptability by the consumers Effect of UV-C light application on liquid food is affected by their turbidity Ineffective when applied to foodstuffs with indefinite shape and structure given its low penetration capacity Huge investment requirements	[3,23,46,50]
	Pulsed light system Rapid disinfection food processing technology Ensure microbial inactivation and at the same time retain the sensory characteristics of foodstuffs Outstandingly short time energy transmission compared to ultraviolet Flexible and eco-friendly procedure In-package treatment	High investment costs Not suitable for application in foods that are opaque and irregularly shaped Possible heating of products due to extended periods of treatment	[3,51]
	Ionizing radiation Minimum effect on quality, taste, appearance, and texture of food Effective in destroying microbes but also insects, mites, and pests Processing time is reasonably shorter comparatively to other technologies Eco-friendly approach leaving no chemicals or residues The dosage applied for food preservation is generally lower and not dangerous for humans after eating irradiated food	If the dose is too high, the functional and the sensory properties of food can be affected Ionizing radiation can be harmful to processors and workers Huge investment costs Problem of consumer acceptance	[3,40,52]
Biological methods	Phage treatment Natural Low cost Self-replicating Commercialization timeframe less stringent than for human therapeutic application High specificity for their target bacterial host with no significant impact on consumers’ resident microbiota No impact on the sensory and quality characteristics of food Ability to infiltrate bacterial biofilms and infect biofilm-embedded bacteria	Sometimes a high phage titer is required for the success of treatment Phages can be inactivated by extreme environmental factors (extreme pH, temperature, UV irradiation, and low oxygen) Some food matrices limit phage diffusion or interfere with phages, reducing their encounter with host bacteria Food additives can impair phage activity Bacteria can develop phage resistance following repeated exposure	[9,11,53,54,55]

**Table 2:** Phage incorporation in active food packaging.

**Target Bacteria** **(Study)**	**Phages**	**Material(s) and Type of Application**	**Food Products**	**Findings**	
				**Bacteria**	**Phages/Films/Coatings**
*Escherichia coli*					
*Escherichia coli* O157:H7 (*amp::lux*) (C918) [265]	Cocktail of *E. coli* phages (EcoM-AG2, EcoM-AG3, and EcoM-AG10)	Modified cellulose membranes with polyvinylamine (positively charged cellulose membranes)	Raw beef	In vitro (bioluminescent assays at 25 °C): At higher phage concentrations (10^9^, 10^7^, and 10^5^ PFU/mL)-similar results obtained with modified and unmodified cellulose membranes with almost complete inhibition; At lower phage concentrations (10^3^): Better results obtained with modified cellulose membranes—after 8 h, the bioluminescence diminished until it reached approximately the same values obtained with higher phage concentrations; Unmodified membranes—bioluminescence patterns are very similar to that of the control.In food: a reduction below the detection limit (aerobic conditions) with at least a 2 log CFU/g reduction at 4 °C for 15 days.	Phage-treated positively charged cellulose membranes showed higher quantities of phage plates than those with the phage-treated unmodified membranes. Drying tests: Desiccated phages irreversibly lost their activity; The siphovirus phage was significantly more tolerant to the effect of air drying than myovirus phages; The addition of maltose or starch significantly improved the tolerance of the T4 phage to air drying; Freeze-drying was the most effective method to dry phages with little decrease in phage activity.
*Escherichia coli* ATCC 11303 [303]	Wild type T4 phage	Core (phage suspension)/shell (poly(ethylene oxide)) fibers	N/A	N/A	Better results obtained with coaxial electrospinning (relatively to simple and emulsion electrospinning)—the incorporated T4 phage totally maintained its activity after several weeks at 4 °C.
*Escherichia coli* [304]	Wild type T4 phage	Core (phage suspension)/shell electrospun fibers made from poly(ethylene oxide), cellulose diacetate, and their blends	N/A	N/A	The phage release rate was dependent on cellulose diacetate/poly(ethylene oxide) ratio and the poly(ethylene oxide) molecular weight. Increasing both parameters resulted in slower phage release.
*Escherichia coli* BL21 [308]	Coliphage T4	Edible whey protein isolate films	Lettuce leaves (release tests)	In vitro: ~5 log difference between the bacterial control and samples containing phage and ~2 log decrease in the samples from the initial inoculation, after 24 h at 22 °C.	The phages Were stable in ambient (22 °C and light) and refrigerated (4 °C and dark) conditions without significant loss in infectivity over a period of 1 month; Were released to a significant degree in an aqueous environment and on a lettuce leaf surface within 3 h; Showed an homogenous distribution within the film matrix.
*Escherichia coli* O104:H4, C1321 [289]	*E. coli* phage cocktail (EcoM-HG2, EcoM-HG7, and EcoM-HG8)	Paper coated with encapsulated phage in alginate beads or Paper impregnated with phage suspension	Alfalfa seed and sprout	At room temperature: Reduction below the detection limit (~5 log CFU/g) after 1 h in the phage-treated (free, encapsulated, or impregnated phage) seeds relative to controls; After 5 days, a reduction in sprouts treated with paper and impregnated with phage or with phage microcapsules of 0.6 and 1.3 log CFU/g, respectively, were observed, in comparison with the free phage results (1.5 log CFU/g).	The phage containing alginate beads (0.5 g containing ~1.22 × 10^10^ PFU) showed a phage release of around 10^7^ PFU/mL and around 10^6^ PFU/mL, after 1 day at 4 °C or 25 °C, respectively. Similar results were obtained after 7 days at 4 °C or 25 °C; No significant difference in antibacterial effect between free and immobilized phages on seeds but on sprouts free phage showed greater results, followed by paper coated with phage microcapsules and paper impregnated with phage.
*Escherichia coli* (EHEC O157:H7 CICC 21530) [301]	*E. coli* O157:H7 phage	Chitosan film containing liposome-encapsulated phage	Beef	Model beef suspension (room temperature with shaking): The antibacterial activity of the chitosan film containing liposome-encapsulated phages was positively associated with its concentration—better results for 400 mg/mL (around 5 log reduction after 7 days in comparison with the bacterial control) were obtained. In food (at 25 °C): The chitosan film containing liposome-encapsulated phage led to a 4.45 log reduction while the chitosan film led to a 0.29 log reduction after 15 days.	The encapsulation efficiency of phages in the liposome was 57.66 ± 0.12%; The chitosan film with a volume ratio of 6:4 (liposome: chitosan) was the most suitable; Phages without liposome protection were unstable and inactive within a short period of time; After liposome encapsulation, the phage inhibitory effect continued for 15 days; No impact of chitosan film embedded with liposome-encapsulated phage on the sensory properties of beef.
*Escherichia coli* O157:H7 [294]	*E. coli* AG10 phages	Pullulan–trehalose films	N/A	N/A	Phage infectivity was maintained for up to 1 month at ambient storage conditions (reduction of 1.90 log PFU/film).
*Escherichia coli* K12 [306]	T4, T5, and T7 phages	Filter paper with phage addition after functionalization with carboxyl methyl cellulose or chitosan	Milk	At 37 °C: All papers with the T4 phage were able to remove *E. coli* from milk; Both functionalized papers removed all *E. coli* (9.3 log CFU/mL) in less than 1 h, while non-functionalized papers only reduced *E. coli* by about 4 log with a total reduction only after 3 h.	All papers extended the lifetime of the infective phage by at least a factor of four, with some papers stabilizing phages for up to one week at 37 °C.
*Escherichia coli* O157:H7 CECT 4076 [299]	vB_EcoMH2W phage	Chitosan-based edible coating	Tomato	A ~3 log bacterial reduction in the samples relative to the controls on tomato surfaces after 6 days at 20 °C.	Phage infectivity was maintained in the film that was applied on the surface of tomatoes for at least 6 days in the presence (higher titer) or absence of *E. coli* at 20 °C.
*Escherichia coli* BL21 [309]	T7 phage	Edible whey protein isolate-based coating	Apples Cherry tomatoes Cucumbers	After 24 h at 4 °C: A ~2 and 4 log bacterial reduction on cut apples and whole cherry tomatoes, respectively, was observed, while no significant reduction was seen for sliced cucumbers.	Films enhanced phage stability during cold storage (4 °C).
*Escherichia coli* ATCC 11303 [305]	Phage T4 (ATCC 11303-B4)	Phage-conjugated indium tin oxide systems	Food components: starch and casein	In vitro, at 37 °C, after 2 h incubation: Upon the 2 h eradication of the ‘1st batch’ (about 4 log reduction), the ‘2nd batch’ of *E. coli* concentration was reduced by around 5 log in just 30 min by all of the indium tin oxide/T4 systems at pH 7; Around 4 log *E. coli* reductionfor all of the indium tin oxide/T4 surfaces at a pH of 7 and 8, after 2 h of incubation; At pH 5, generally no *E. coli* reduction was seen after 2 h of incubation. In vitro (in the presence of food components at pH 7): A 3–4 log bacterial reduction after 2 h was seen, similar to that observed in only medium.	All the indium tin oxide/T4 systems maintained their antimicrobial activity in the presence of model food components (starch and casein), but this activity was still affected by pH.
*Escherichia coli* (DH5α and O157:H7 STEC strains) [307]	Cocktail of T-even type phages (DT1 to DT6)	Whey protein concentrate films	Meat	In vitro (growth inhibition assay): At 4 and 37 °C— A reduction to non-detectable levels for DH5α and O157:H7 STEC strains, after 24 h was seen; At 24 °C—both strains grew; however, the extent of growth was lesser than in the controls.In vitro (inhibition zone assay): At 4 °C, a 1.5 ± 0.1 mm of zone of inhibition for the films with added phages compared to film disk without phages (no inhibition) after 1 week = 1 month. In food: Total elimination (~2 log) of both DH5α and STEC strains of *E. coli* in the meat stored for 24 h at 4 °C and 1 h at 37 °C.	Phage cocktail embedded in the films were stable within 5 weeks of storage (better results at 4 °C than at 24 °C).
*Escherichia coli* CECT 434 [298]	*Salmonella* phage φ135 and *E. coli* phage vB_EcoS-EC4 (EC4)	Phages and cinnamaldehyde incorporated on sodium alginate emulsion-based films	N/A	In vitro: Better results with a combination of both phages at a higher concentration of cinnamaldehyde (0.4%), with a reduction until reaching the detection limit of the method (~7 log) after 24 h at 20 °C.	The incorporation of phages into the film did not introduce significant changes to the film characteristics, unlike cinnamaldehyde, which increased the roughness, thickness, and swelling ability of films.
*Escherichia coli* K12 (ATCC 23724) and O157:H7 H1730 [300]	Phage T4 (ATCC 11303)	Polycaprolactone films (phage addition by physical adsorption or chemical functionalization)	Raw beef	*In vitro E. coli* K12 reduction with chemically functionalized film compared to films with no phages: 37 °C—maximum reduction between 3 and 5 h to about 7 log and 2.44 log after 120 h; 20 °C—around 6 log maximum bacterial reduction after 24 h and around 2 log after 120 h; 10 °C—maximum decrease of around 3 log after 120 h; 4 °C—no growth of *E. coli* and no antibacterial activity in the samples treated with films was observed.In food: chemically functionalized film reduced *E. coli* O157:H7 to ~2 log after 120 h at 10 °C.	Chemically functionalized film showed more antibacterial efficacy than the physically adsorbed film.
*Escherichia coli* O157:H7 (EHEC O157:H7 CICC 21530) [302]	*E. coli* O157 phage	Co-encapsulation of phages and D-phenylalanine into sodium alginate/polyethylene oxide nanofibers based-films	Beef Cherry tomatoes Cucumber	In vitro (37 °C): There was a decrease of about 3 log in planktonic *E. coli* after 8 h; An around 7 log reduction in the *E. coli* biofilm on stainless steel after 72 h was seen. In food (after 4 days): Beef—reduction of ~2 log of planktonic *E. coli* at both 4 and 25 °C; Cucumber—reduction of ~3 and 2 log of *E. coli* biofilm at 4 and 25 °C, respectively; Cherry tomatoes—reduction of ~2 and 1.5 log on the *E. coli* biofilm at 4 and 25 °C, respectively.	The films showed good mechanical properties and thermal stability; Phages were able to remain viable in nanofiber films with high release rate; The addition of D- phenylalanine significantly enhanced the antimicrobial activity of the nanofiber films.
*Salmonella* spp.					
*Salmonella enterica* ser. Typhimurium [314]	Phage cocktail (BFSE16, BFSE18, PaDTA1, PaDTA9, PaDTA10, and PaDTA11)	Cellulose acetate films	N/A	In vitro (diffusion in liquid medium): Increase in the lag phase and slower growth of microorganisms in the sample containing incorporated phages in the films, compared to control, at 150 rpm at 35 ± 2 °C. In vitro (diffusion in solid medium): Halos of films containing phages of 1.23–1.35 cm, larger than that of the pure cellulose acetate film (1 cm), at 35 ± 2 °C for 24 h.	The mechanical and physical properties of films (thickness, elongation, and puncture resistance) showed no significant differences after phage addition; The addition of phage altered the film surface, reducing the transparency, tensile resistance, and the modulus of elasticity of the film and increased the porosity; Phages remained viable for 14 days at 23 ± 2 °C and at a relative humidity 50 ± 10%, and were no longer detected after that time.
*Salmonella* sp. cocktail (*S.* Typhimurium DT104, 19485A96 SGI1 and ATCC 13311, *S.* Heidelberg ATCC 8326, *S.* Enteritidis ATCC 4931 and *S.* Newport ATCC 6962) [312]	*Salmonella* phage Felix O1	Poly(lactic acid) films with a xanthan coating containing phage	Sliced turkey	In vitro (microtiter plate assay): At 37 °C, *S.* Typhimurium DT104 cultures showed significantly slower growth and lower final bacterial density compared to the control, while at 25 °C, *S.* Typhimurium was reduced in a similar way in the presence and absence of phages over a period of 20 h. In food: Better results under anaerobic packaging with reductions in Salmonella sp. cocktail in about 0.832 and 1.30 log at 4 °C and 10 °C after 30 days.	99.9% of phage released within 30 min into meat.
*Salmonella* Newport [294]	*Salmonella* CG4-1	Paper coated with pullulan–trehalose mixture containing phages or Pullulan–trehalose films incorporating phages	N/A	In vitro, a 4.59 log CFU/cm^2^ bacterial reduction at ambient conditions (∼22–25 °C) after 1 month was observed with paper coated with a pullulan–trehalose mixture and containing phages.	Phage infectivity was maintained for up 2 months in the pullulan–trehalose films in ambient storage conditions.
*Salmonella* Enteritidis EX2 [298]	*Salmonella* phage φ135 and *E. coli* phage vB_EcoS-EC4 (EC4)	Phages and cinnamaldehyde incorporated on sodium alginate emulsion-based films	N/A	In vitro, better results with a combination of both phages at the higher concentration of cinnamaldehyde (0.4%) were obtained, with reduction until the detection limit of the method (~8 log) was reached after 24 h at 20 °C.	The incorporation of phages into the film did not introduce significant changes in its characteristics, unlike cinnamaldehyde, which increased the roughness, thickness, and swelling ability of films.
*Salmonella* Enteritidis (H40499 SDE) [319]	*Salmonella* Enteritidis phage Felix O1	Phage incorporation into polyvinyl alcohol coatings and fibers deposited by casting and electrospinning on polyhydroxybutyrate/poly-hydroxyvalerate films	N/A	N/A	Polyvinyl alcohol increased the moisture content, the solubility, and the hydrophilicity of the films; Phages were successfully incorporated and remained viable (10^6^ PFU/mL) after the formation of the coating and nanofibers.
*Salmonella* mixture [315]	Phage cocktail: *S.* Enteritidis F5–4, *S.* Typhimurium L2–1, and *S.* Typhimurium ICB1–1	Phage-based edible coatings of whey protein concentrate, carboxymethyl cellulose, chitosan, or sodium alginate	Strawberries	The largest antimicrobial effect was observed with a whey protein concentrate coating, with a reduction of 3.1 log CFU/g after 5 days at 4 °C, compared to the other tested polymers.	For a period of 5 days at 4 °C: Whey protein concentrate coating showedthe smallest escalation in pH and the smallest decrease in the titratable acidity of dip-coated strawberries; During storage, a 0.7 log-unit (PFU/g) reduction in whey protein concentrate coating was observed compared to the phage reduction observed with other polymers (around 1.0).
*Salmonella* Enteritidis (ATCC 13076) [320]	*Salmonella* Enteritidis phage PBSE191 (BP-1370)	Polyvinyl alcohol film	Chicken eggshell	In vitro: Significant bacterial reduction (2 × 10^5^ CFU/film within 2 h) in the phage-containing films compared to the bacterial control-containing film without phages, at 37 °C. On chicken eggshell surface: An about 2 log CFU reduction within 24 h at 5 °C and 50% relative humidity was observed.	Phages remained stable and were effectively released from the polyvinyl alcohol film without any substantial loss in the phage titer at 5 °C and at 50% relative humidity for 30 days.
*Salmonella* Enteritidis (ATCC 13076) [311]	*Salmonella* Enteritidis phage PBSE191 (BP-1370)	κ-carrageenan (KC) and konjac glucomannan (KGM) hydrogel film containing adsorbed phages	Raw chicken meat	Around 1 log CFU/mL reduction within 3 h at 25 °C and within 48 h at 5 °C.	KC/KGM-based hydrogel with a ratio of 7:3 showed the highest compressive strength; 40% sorbitol containing KC/KGM hydrogel film showed the highest tensile strength; Phage adsorption significantly increased the tensile strength and water swelling ratio and decreased water solubility and water vapor permeability; Phage adsorption did not change the chemical structure of the films; The phage-adsorbed film had an overall smooth and layered morphology.
*Salmonella enterica* CCCD-S004 [316]	Phage cocktail (SentS01L and SentS01T phages)	Sodium alginate edible coating	Ripened cheese	Coating containing phage cocktail was removed from a cheese matrix sample and positioned in the center of a bacterial lawn of *Salmonella enterica* CCCD-S004. After incubation (24 h at 37 °C), the presence of clear zones of lysis in the bacterial lawn surrounding the coating was observed.	The antibacterial coating showed adequate physicochemical characteristics and zero cytotoxicity in HaCaT and 3T3 cell lines.
*Salmonella* Enteritidis (MET S1–001) [313]	Phage MET P1-001_43	Layer-by-layer assembly of chitosan/alginate thin films incorporating phages	Chicken meat	Wrapping a *S.* Enteritidis-contaminated chicken piece with aluminum foil whose surface was modified with phage-loaded chitosan/alginate multilayers reduced bacterial numbers in more than 1.0 log CFU/cm^2^. Similar results were obtained with free phages.	The phage-loaded chitosan/alginate multilayers showed antibacterial activity at pH 7, but not in acidic conditions; Surface roughness decreased distinctly upon treatment of multilayers with phage-containing NaCl solution.
*Listeria monocytogenes*					
*Listeria monocytogenes* C391 [265]	Cocktail of *Listeria* phages (LinM-AG8, LmoM-AG13, and LmoM-AG20)	Modified cellulose membranes with polyvinylamine (positively charged cellulose membranes)	RTE oven-roasted turkey breast	Better results obtained at 4 °C and in vacuum conditions: *Listeria* reduction of 4 log CFU/g after 15 days.	Immobilized phages were able to control the growth of *L. monocytogenes* in meat incubated at different temperatures and under different packaging conditions.
*Listeria monocytogenes* cocktail (Li0512, Li0529, ATCC19115 and 08–5578, serotype 1/2b,1/2a, 4b and 1/2a, respectively) [289]	LISTEX™ P100 phage (RTE turkey) or *L. monocytogenes* phage cocktail (LinM-AG8, LmoM-AG13, and LmoM-AG20) (freshly cut cantaloupe)	Modified cellulose membranes with polyvinylamine (positively charged cellulose membranes)	RTE turkey Fresh cut cantaloupe	RTE turkey: Significant *L. monocytogenes* reduction at 4 °C and 10 °C, with around 1 log and 2 log CFU/cm^2^ reduction, respectively, after 5 days. Fresh cut cantaloupe: Reduction of ~1 log at the end of different storage conditions (4 and 12 °C for 5 days, and 25 °C for 24 h) in the samples containing the immobilized phage compared to controls; Better results were obtained using free phages with reductions of 3, 4, and 3 log at 4 °C (after 5 days), 12 °C (after 5 days), and 25 °C (after 24 h), respectively.	RTE turkey: No significant difference in antibacterial effect between free and immobilized phages. Fresh cut cantaloupe: Spray-coating free phages showed greater antibacterial effect than immobilized phages.
*Listeria monocytogenes* cocktail (FSL F6-367, ATCC 19115, 08e5578, C6-0003, LI 0512) [312]	*Listeria* phage A511	Poly(lactic acid) films with a xanthan coating containing phages	Sliced turkey	In vitro (microtiter plate assay): At 37 °C, a significant decrease in final cell density in the grown cultures of *L. monocytogenes* ATCC 19115 in the presence of the films containing phage, relative to the controls, after 3 h at 25 °C, was observed, with a significant reduction only seen after 21 h. In food: Reductions in *L. monocytogenes* cocktail of 6.31 log at 4 °C and 1.52 log at 10 °C, in anaerobic packaging after 30 days, and reductions of 3.79 log at 4 °C and 2.17 log at 10 °C, after 14 days, in aerobic packaging.	99.9% of both phages were successfully released from the film within 30 min onto the meat samples.
*Listeria monocytogenes* serotype 1/2a [294]	LISTEX P100	Paper coated with pullulan–trehalose mixture containing phages or Pullulan–trehalose films incorporating phages	N/A	In vitro, more than 2 log CFU/cm^2^ reduction at ambient conditions (∼22–25 °C) after 6 weeks, with paper coated with pullulan–trehalose mixture containing phages was observed.	Phage infectivity was maintained for up to 2 months in the pullulan–trehalose films at ambient storage conditions.
*Listeria monocytogenes* serotype 1/2a [321]	LISTEX P100	Phage incorporated into pullulan–trehalose films	N/A	N/A	Vacuum drying and storing in enclosed containers enhanced the long-term viability of the phage by over 1000-fold; Main cause of titer reduction in the film was exposure to high humidity.
*Listeria monocytogenes* (CECT 934, ATCC 19114) [288]	LISTEX™ P100 phage	Films of (1) sodium caseinate, (2) sodium alginate mixed with gelatine, and (3) polyvinyl alcohol	N/A	All films showed *in vitro* antimicrobial capacity of close to 1 log after 24 h at 30 °C; The effectiveness of polyvinyl alcohol films was greater at 8 °C, reaching a 2 log reduction after 8 days.	The incorporation of phages did not alter the morphology, color, opacity, or thermal stability of the films; Better antibacterial results were obtained with free phage compared to incorporated phages.
*Listeria monocytogenes* (ATCC 19113) [318]	*Listeria* phage A511	Whey protein concentrate (WPC), pullulan (PULL) films, and their blends	N/A	In vitro: Inhibition assay by disk diffusion—30WPC:70PULL showed a significant reduction in inhibitory effect after 60 days compared to 50WPC:50PULL and 70WPC:30PULL, which completely lost their inhibitory effects after 60 and 40 days, respectively, at 30 °C; Growth inhibition assay with 30WPC:70PULL film—4–5 log of *L. monocytogenes* reduction after 24 h at 25 °C.	Pure films of WPC and PULL were not successful in phage stabilization; Higher phage recovery in the 30WPC:70PULL film; Phage distribution in the films was uniform and resulted in enhanced opacity; In the 30WPC:70PULL film, the elastic modulus and tensile strength were enhanced while the elongation at break diminished after 60 days; The film 30WPC:70PULL had the best physical, mechanical, and anti-*Listeria* performance during storage (60 days at 25 °C and at a relative humidity of 53%).
*Listeria monocytogenes* (ATCC 19113) [310]	*Listeria* phage A511	Bilayer films of whey protein concentrate/pullulan (WP) containing phage and poly (lactic acid) (PLA)	Chicken breast	In vitro: Disk diffusion assay—The diameter of the inhibition zone in the bilayer and monolayer films were similar and did not differ significantly at 30 °C; Growth inhibition—The growth of *L. monocytogenes* in the phage-containing film treatment was significantly lower than that in the phage-free film treatment (around 5 log reduction), after 24 h at 25 °C. In food: the phage-containing films inhibited *Listeria* in chicken breast filets in a similar way to free phages with at least a 1.5 log CFU/cm^2^ reduction at both 4 and 10 °C after 120 h compared to the control-containing film without phages.	The addition of 30% thickness ratios of PLA to WP film enhanced the mechanical, barrier, and visual properties of bilayer film; Among the bilayer films, 30PLA/70WP was the best film, with the highest phage recovery and phage stability; This film showed a shelf life of 20 days at 25 °C and 50% relative humidity.
*Listeria* *monocytogenes* [317]	ATCC A511 phage	Whey protein isolate (WPI)-based coating	Cheese	At the end of storage period (16 days at 4 °C), phages added to water or to WPI reduced bacterial counts in 0.39 and 0.86 log CFU/g, respectively.	Phages remained stable in the WPI coating for 16 days at 4 °, and had a similar stability to phages in buffer; A511 phage remained stable on the surface of WPI dip-coated cheese in the presence of *L. monocytogenes*; In the absence of *L.* *monocytogenes*, the phage concentration was reduced by 0.8 log PFU/g; The phage–WPI coating had a significant effect on the color, hardness, and springiness of cheese.
Other bacteria					
*Vibrio parahaemolyticus* ATCC 17802 [322]	Phage isolated from raw bonito fishes (*Sarda sarda*)	Edible methylcellulose films coated with capsules of sodium alginate containing phages	Raw fish filets	In vitro (after 24 h at room temperature): 1.27 and 3.99 log of bacterial reduction in the treated samples with film containing phages compared to the initial bacterial inoculum and the bacterial control, respectively. In food (after 14 days at 4 °C): A 2.65 and 6.46 log reduction in bacteria in the treated samples with films containing phages compared to the initial bacterial inoculum and the bacterial control, respectively.	Phage stability: In both tested conditions (darkness at 4 °C and illuminated room at 22 °C), the most significant decrease in the encapsulated phage stability was observed on the 7th and 14th days; Lower phage stability at 22 °C in a lit environment with a decrease of 4 log after 14 days. Phage release from the film into water: 1.5 × 10^5^ PFU/mL during the first 30 min and in total 1.6 × 10^7^ PFU/mL after 5 h of incubation.
*Pseudomonas fluorescens* PF7A [323]	Phage ϕIBB-PF7A	Sodium alginate-based films	Chicken breast filet	In vitro (at 4 °C): A significant decrease in *P. fluorescens* growth in films that incorporated phages (1.9 and 4 log reductions after 24 h) when in direct contact with *P. fluorescens* or when completely immersed on a bacterial suspension, respectively. In food (at 4 °C): Films incorporating phages reduced *P. fluorescens* counts by 2 log after 2 days and maintained a significant reduction for the next 5 days (1 log).	Phages were homogeneously distributed inside the films; A decrease in phage viability by ~2.4 log was detected after 8 weeks at 4 °C, while phages were inactivated after 15 s and 30 s of exposure at 77.5 and 67.5 °C, respectively.
*Clavibacter michiganensis* subsp. *nebraskensis* (Cmn-91R) [324]	Phage CN8	Coatings based on phages, polymers (polyvinylpyrrolidone, polyvinyl alcohol, or poly(methyl vinyl ether)), and stabilizers (whey protein isolate, skim milk, sucrose, maltodextrin, or D-mannitol)	Maize seeds	Coating of polyvinyl alcohol combined with whey protein isolate containing phage significantly reduced target bacterial cells up to 3.8 × 10^3^ CFU/seed after four months at 10 °C, without affecting seed germination.	Polyvinyl alcohol offered the greatest stability for CN8 phages on seeds when coatings did not contain a stabilizer; Polyvinyl alcohol combined with whey protein isolate stabilizer maintained CN8 phage activity for seven months and four months at 10 and 26 °C, respectively.
*Staphylococcus aureus* IPLA1 [325]	Phage phiIPLA-RODI	Edible gelatine films/coatings	Cottage cheese	In vitro: Reductions of 5 and 7 log for the films with the lowest and the highest phage concentrations, respectively, after 17 h at 37 °C and at 250 rpm. On cheese (at 4 °C): A lower reduction than that obtained *in vitro* (1–2 log) was observed; Best results (except for the highest phage concentration) were observed when the cheese was immersed in the film-forming solution containing phages and the coating was directly formed on the surface of the cheese.	The edible films/coatings were not physically impacted by the addition of phages.

N/A—not available; KC—κ-carrageenan; KGM—konjac glucomannan; RTE—ready-to-eat; WPC—whey protein concentrate; PULL—pullulan; WP—whey protein concentrate/pullulan; PLA—poly (lactic acid); WPI—whey protein isolate.

## Data Availability

No new data were created or analyzed in this study.

## Supplementary Materials

The following supporting information can be downloaded at: https://www.mdpi.com/article/10.3390/microorganisms13030515/s1. Table S1: Results of recent studies on the application of free phages directly to food.
