# Supplementary material for: A Review on Recent Trends in Bacteriophages for Post-Harvest Food Decontamination
Source: Microorganisms. 2025 Feb 27;13(3):515. doi: 10.3390/microorganisms13030515 (PMC11946132; doi:10.3390/microorganisms13030515)
Supplement: Supplementary file 1 [file microorganisms-13-00515-s001.zip › microorganisms-3457244-supplementary.pdf]

Supplementary Table S1. Results of recent studies on the application of free phages directly to food.

Bacterial strain / Type of application	Phage / Type of application	Tested food	Outcomes – Bacterial reduction	Study Reference
***Salmonella* spp.**				
S. Enteritidis ATCC 13076<br><br>Pipetting:<br>- 10^4 CFU/cm^2<br>- room temperature for 10 min	Phage vB_SalM_SPJ41<br><br>Pipetting - 10^8 PFU/cm^2	Lettuce and salmon	Lettuce:<br>- 4 ºC - ≈3.0 log CFU/cm^2 reduction after 3 h;<br>- 25 °C - 3.2 log CFU/cm^2 decrease after 24 h.<br><br>Salmon:<br>- 4 ºC - reduction of ≈ 2.2 log CFU/g was obtained in the treated group compared to the control, after 24 h;<br>- 15 ºC - decrease below the detection limit of the method (50 CFU/g) for 6-12 h. After 24 h, 3.9 log CFU/g decrease.	(T. Li et al., 2023)
S. Enteritidis PTCC 1787<br><br>Pipetting - liquid whole egg (10^6 CFU/mL)<br><br>Immersion - whole and unbroken eggs (10^6 CFU/mL) - 30 min before being placed to dry	Phage Rostam<br><br>Pipetting - liquid whole egg. Different MOIs (10 and 10,000)<br><br>Immersion - whole and unbroken eggs immersed in a phage container (10^9 PFU/mL) for 5 s and allowed to dry for 30 min	Liquid whole egg and eggshell	Liquid whole egg:<br>- 4 ºC - decrease under the detection limit of the method (4.0 log CFU/mL reduction) for both values of MOI 10,000 (after 24 h) and 10 (after 48 h), maintained for 5 days.<br>- 25 ºC, highest reduction of 4.5 and 1.8 log CFU/mL at MOI 10,000 and 10, respectively, after 5 days.<br><br>Eggshells:<br>reduction under the detection limit of the method at 4 ºC (after 24 h) and 25 ºC (after 48 h).	(Azari et al., 2023)
Bacterial cocktail of *S. enterica* subsp. *enterica* serotypes Enteritidis, Typhimurium, and Kentucky<br><br>Pipetting (10^7 CFU/ mL) – 30 min at room temperature	Phage cocktail (phages SE4, SE13, and SE20)<br><br>Spraying (10^9 PFU/mL) - MOI of ≈1,000	Raw chicken breast	- 10 ºC - bacterial counts were reduced by more than 3.2 log compared to the control, after 5 days;<br><br>- 22 ºC - bacterial reduction of more than 1.7 log after 16 h of storage.	(Brenner et al., 2024)

Escherichia coli				
Enterohemorrhagic *E. coli* O157:H7 EO157-1<br><br>Pipetting (10^4-10^5 log CFU/mL)	Phage SQ17<br><br>Pipetting (10^8 log PFU/mL) - MOI values of 10^3-10^4	Milk, raw beef and lettuce	Milk:<br>4 ºC:<br>- reduction below the detection limit of the method was obtained in both skim and whole milk with ≈4.7 log CFU/mL (after 4 h) and 4.5 log CFU/mL reduction (after 8 h), respectively;<br>- no regrowth after 7 days.<br>25 ºC:<br>- reduction of ≈4.0 log CFU/mL after 4 h;<br>- bacterial regrowth after 12 and 24 h in both skim and whole milk.<br><br>Raw beef:<br>4 ºC:<br>- reduction by 2.4 log CFU/piece after 12 h;<br>- no regrowth after 5 days;<br>25 ºC:<br>- reduction of 0.7 log CFU/piece after 4 h;<br>- bacterial concentration of the phage-treated sample close to the control after 48 h.<br><br>Fresh lettuce:<br>- reduction by 2.2 log CFU/piece and 3.8 log CFU/piece after 12 h at 4 ºC and 25 ºC, respectively;<br>- no bacterial regrowth for both temperatures.	(Y. Zhou et al., 2022)
E. coli O157:H7 C7927<br><br>Pipetting (10^5 and 10^6 CFU/mL)	Phage CAM-21<br><br>Pipetting (10^9 PFU/mL)	Milk, ground beef and baby spinach	4 ºC:<br>- Milk - decrease of 1.4 and 2.0 log CFU/mL at MOI values of 1,000 and 10,000, respectively, after 24 h;<br><br>- Ground beef - MOI of 1,000 and 10,000 led to bacterial reductions of 1.4 and 1.3 log CFU/g, respectively, after 24 h;	(Choo et al., 2023)

			- Baby spinach - MOI of 1,000 and MOI of 10,000 led to bacterial reductions of 1.3 and 1.4 log CFU/g, respectively, after 24 h.	
E. coli O157:H7 ATCC 43894 and ATCC 43888<br><br>Immersion (10^6 CFU/mL) for 30 min and 1 h of storage at room temperature	Phages PECP14 and PECP20<br><br>Immersion (10^8 PFU/mL) for 15 min	Radish sprouts	4 ºC:<br>- *E. coli* O157:H7 ATCC 43894 - significant 0.9 log-reduction after 1 h of PECP14 treatment, compared to the phage untreated control group, was maintained up to 48 hours;<br>- *E. coli* O157:H7 ATCC 43888 - a significant reduction of 1.2 log CFU/g after 1 h of PECP20 treatment.<br><br>25 ºC:<br>- *E. coli* O157:H7 ATCC 43894 - significant 0.9 log-reduction after 1 h with the phage PECP14;<br>- *E. coli* O157:H7 ATCC 43888 - reductions of 1.6 and 1.0 log CFU/g after 12 and 24 h of PECP20 treatment, respectively.	(Oh et al., 2024)
Listeria monocytogenes				
L. monocytogenes strains (10403S, FSL R9-5621, FSL R9-5623, FSL R9-5624, or FSL R9-5625)<br><br>Pipetting with 1 of the 5 strains (10^5 log CFU/g) after 30 min of incubation at room temperature	Phage cocktail ListShield™<br><br>Pipetting (8 × 10^6 PFU/g)	Laboratory cheese model	- At 22 ºC, bacterial counts were significantly lower, with the better results for FSL R9-5621 and FSL R9-5624 (after 1 day), and FSL R9-5625 (after 14 days) with at least 2.0 log-reduction, than those on phage-treated cheese stored at 6 or 14 ºC;<br>- Better bacterial reductions were obtained on cheese made at higher pH (6.0 and 6.5) compared to results on cheese made at pH 5.5, where *L. monocytogenes* did not grow;<br>- For the specific strain of *L. monocytogenes* FSL R9-5624, reductions of ≈ 2.0, 1.0 and 0.5 log CFU/g for pH values of 6.5, 6.0 and 5.5, respectively.	(Henderson et al., 2019)
L. monocytogenes cocktail (ATCC 19111 and ATCC 19115)	Phage cocktail (phages LMPC01, LMPC02, and LMPC03) – MOI 10	Celery and enoki mushroom	Reduction of 2.2 and 1.8 log CFU/g of *L. monocytogenes* in celery and enoki mushroom, respectively, stored at 4 ºC for 7 days.	(Byun et al., 2022)

Immersion (10^4 CFU/mL) and storage for 1 h for attachment at 4 ºC and drying for 10 min	Immersion for 30 min			
Biofilms of *L. monocytogenes* cocktail (ATCC 19111 and ATCC 19115)<br><br>Immersion (10^4 CFU/mL) and 1 h storage at 4 ºC and drying for 10 min	Phage cocktail (phages LMPC01, LMPC02, and LMPC03) – MOI 100<br><br>Immersion for 30 min	Celery and chicken meat	4 ºC:<br>- Celery - 1.6 log maximum reduction after 5 days.<br><br>- Chicken meat - 0.5 log maximum reduction after 7 days, by the phage comparatively to the untreated control group.	(Byun et al., 2024)
***Campylobacter* spp.**				
C. jejuni strain CF84<br><br>10^4 CFU/mL – 4 h incubation at 4 °C	Phage CJ01<br><br>Spraying (10^6 PFU/mL) – MOI of 100	Mutton and chicken meat	Reductions of ≈1.7 log CFU/g in both mutton and chicken meat, after 48 h at 4 ºC.	(Thung et al., 2020)
Multidrug-resistant *C. coli* Cc512<br><br>10^6 CFU/mL	Phage CP6<br><br>Pipetting (10^7−10^9 PFU/g)	Raw chicken meat	Reductions of 1.0 (10^8 and 10^7 PFU/mL) to 1.2 log (10^9 PFU/mL), after 6 h, at 4 ºC, with a slight decrease after 24-36 h.	(Xiaoyan Zhang et al., 2024)
